# Fungal Indicators of Sensitivity and Resistance to Long-Term Maize Monoculture: A Culture-Independent Approach

**DOI:** 10.3389/fmicb.2021.799378

**Published:** 2022-01-03

**Authors:** Agnieszka Wolińska, Jacek Podlewski, Andrzej Słomczewski, Jarosław Grządziel, Anna Gałązka, Agnieszka Kuźniar

**Affiliations:** ^1^Department of Biology and Biotechnology of Microorganisms, Institute of Biological Sciences, The John Paul II Catholic University of Lublin, Lublin, Poland; ^2^Potulicka Foundation Economic Center, Wojnowo, Poland; ^3^Department of Agriculture Microbiology, Institute of Soil Science and Plant Cultivation in Pulawy, Puławy, Poland

**Keywords:** fungal community, maize monoculture, intercropping mixture, precise farming, fungal indicators

## Abstract

Although fungi are regarded as very important components of soils, the knowledge of their community in agricultural (monocultural) soils is still limited. This indicates that soil fungal communities are investigated less intensively than bacteria. Therefore, the main goal of this paper was to evaluate the fungal mycobiome structure in monoculture soils in a culture-independent approach. Firstly, the study was conducted to identify the core mycobiome composition and its variability at different stages of the maize growing season (spring, summer, and autumn). Secondly, we identified and recommended fungal indicators of both sensitivity and resistance to long-term maize monoculture. Two neighboring fields from the Potulicka Foundation area were selected for the study: K20 sown with a Gorzow mixture (intercropping mixture) to improve soil quality after a maize monoculture in 2020 and K21, where long-term (over 30 years) monoculture cultivation was continued. The basic chemical features [acidity, redox potential, total organic carbon (TOC), and moisture] of soils were determined, fungal genetic diversity was assessed by ITS next generation sequencing (NGS) analyses, and biodiversity indices were calculated. The results of the NGS technique facilitated recognition and classification of the fungal mycobiome to the taxonomic genus level and changes in the fungal structure in the three periods (spring, summer, and autumn) were assessed. It was evidenced that the mycobiome composition was dependent on both the seasons and the agricultural practices. It was also found that even a 1-year break in the monoculture in favor of an intercropping mixture improved soil properties thus contributing to higher biodiversity. *Mortierella* was recommended as a potential indicator of sensitivity to long-term maize cultivation, whereas *Solicoccozyma* and *Exophiala* were proposed as indicators of resistance to long-term maize cultivation. We proved that the precision farming principles applied on the Potulicka Foundation farm had a very positive effect on fungal biodiversity, which was high even in the long-term maize monoculture field. Therefore, the monoculture cultivation carried out in this way does not induce biological degradation of monoculture soils but preserves their good biological quality.

## Introduction

Fungi are considered as critical and very important components of soil ecosystems (Ellouze et al., 2014; Yang et al., 2017; Frąc et al., 2018), as they are the primary decomposers of soil complex compounds, i.e., lignocelluloses (He et al., 2017; Choudhary et al., 2018) and provide ecological services influencing the production of food and bioproducts (Ellouze et al., 2014). Fungi not only convert dead organic matter into biomass, organic acids, and carbon dioxide but also have the ability to sorb and accumulate toxic metals in their organisms (Frąc et al., 2018). An important fungal group is arbuscular mycorrhizal fungi (AMF) establishing symbiotic relationships with the roots of most crops, i.e., wheat, barley, triticale, maize, rice, soybean, etc. (An et al., 2010; Ellouze et al., 2014; Jamiołkowska et al., 2017). AMF is also crucial for the proper bilateral exchange of carbon (C) and phosphorus (P) and the production of glomalins, i.e., unique fungal glycoproteins (Jamiołkowska et al., 2017; Gałązka and Grządziel, 2018; Furtak et al., 2021). However, fungal diversity in the soil environment can be limited by several environmental elements, i.e., tillage, crop rotation, and biotic and abiotic factors (Rouphael et al., 2015; Frąc et al., 2018; Gałązka and Grządziel, 2018; Jamiołkowska et al., 2018). Importantly, fungi influence the function of the whole ecosystem through interactions with other soil organisms (Yang et al., 2017; Jamiołkowska et al., 2018; Furtak et al., 2021). It is worth mentioning here that the knowledge of fungal communities in agricultural soils is still limited (Rousk et al., 2010; Orgiazzi et al., 2012; Wang et al., 2016; Gałązka and Grządziel, 2018), which indicates that soil fungal communities are investigated less intensively than bacteria.

Studies of fungal diversity and community structure have been greatly limited by the problems with culturing (it was estimated that <5% fungi are culturable) and morphological identification (He et al., 2017; Yang et al., 2017). However, in recent years, the development of culture-independent techniques has made it possible to identify both bacteria and fungi representing a group of viable but non-cultivable microorganisms (Orgiazzi et al., 2013; Frąc et al., 2018; Wolińska et al., 2018, 2020; Grządziel and Gałązka, 2019). Based on the next generation sequencing (NGS) of the Internal Transcribed Spacer (ITS) region, Gałązka and Grządziel (2018) reported Zygomycota, Basidiomycota, and Ascomycota to be the dominant fungal phyla in maize monoculture. Among fungal genera present in these soils, the highest relative abundance was identified for representatives of *Penicillium*, *Geomyces*, *Mortierella*, and *Pseudogymnoascus* (Gałązka and Grządziel, 2018). However, apart from the study conducted by Gałązka and Grządziel (2018), no similar papers have described the fungal mycobiome structure in Polish maize long-term monocultures, which indicates the validity of the present research.

It is worth emphasizing that monocultures of different crops are usually regarded as environments with low fertility, productivity, quality, and biodiversity. There is also a view that monoculture soils are therefore not very interesting and not worth investigating. Some authors have proved that the properties of long-term monocultures undergo biological degradation (Liang et al., 2011; Gałązka et al., 2017; Han et al., 2017; Wolińska et al., 2018; Bojarszczuk et al., 2019). It is an important problem because low-quality soils are usually exploited as monocultures involving long-term cultivation of plants of a single species with similar soil requirements in the same area, which causes rapid sterilization and changes in the structure of these soils (Liang et al., 2011; Gałązka et al., 2017; Choudhary et al., 2018; Bojarszczuk et al., 2019). Consequently, it is strongly advisable to assess the current state and microbiological quality of monoculture soils to be able to react instantaneously to any potentially unfavorable changes that may be associated with a decline in their biodiversity.

Pervaiz et al. (2020) predicted a hypothetical impact of continuous cropping (monoculture) on soil health. Considering the complexity of the process of selecting appropriate indicators, at the beginning they were divided into biotic and abiotic indicators of soil health. The biotic indicators may include but are not limited to total biomass, activities, functioning, community composition, and interactions of soil-inhabiting macroorganisms and microorganisms that determine the trophic or food-web complexity of soil ecosystems (Pervaiz et al., 2020; Behnke et al., 2021; Beule and Karlovsky, 2021; Neupane et al., 2021). The abiotic indicators of soil health include but are not limited to soil aggregation, aggregate stability, organic C and organic matter contents, nutrient cycling and sequestration, the composition of soil exudates and metabolites, nutrient balance, and other essential properties such as pH and CEC (Pervaiz et al., 2020; Behnke et al., 2021). Burke et al. (2019) revealed that pH, P, and the CN ratio were the strongest predictors shaping fungal communities.

According to the estimates reported by the Polish Corn Growers Association, the acreage of corn production in Poland in 2020 was about 2 million hectares. This means that maize is one of the most popular plants in Poland cultivated as a monoculture. Maize cultivation in monoculture is a common practice in many countries in the world (Gałązka et al., 2018). Consequently, identification of the biodiversity of non-cultivable fungi present in maize monocultures will therefore fill the existing gap in the knowledge of the fungal structure in soils that are commonly regarded as low quality and fertility. It may also verify the knowledge of the actual quality of maize monocultures.

We hypothesized in the current paper that, given the biological equilibrium in nature, it should be possible to select fungal indicators showing both sensitivity and resistance to long-term maize monoculture. To reinforce the final inference, fluctuations in the fungal mycobiome structure were analyzed in three terms of the maize growing season: in spring (before maize sowing), in the middle of vegetation (summer), and at the end of the growing season (autumn, after maize harvest). Consequently, indicators of either sensitivity or resistance to long-term monoculture crops were recommended based on either a decrease or an increase in the relative abundance of fungal genera, with a given relationship having to persist over the three terms of the maize growing season.

The main efforts in the present study were focused on the assessment of the fungal diversity structure with the use of a culture-independent approach in agricultural monoculture soils. Firstly, the research was conducted to identify the core mycobiome composition and its variability at different stages of the maize growing season. Secondly, the aim was to identify and recommend fungal indicators of both sensitivity and resistance to long-term maize monoculture.

## Materials and Methods

### Study Site and Soil Sampling

**Figure 1:**
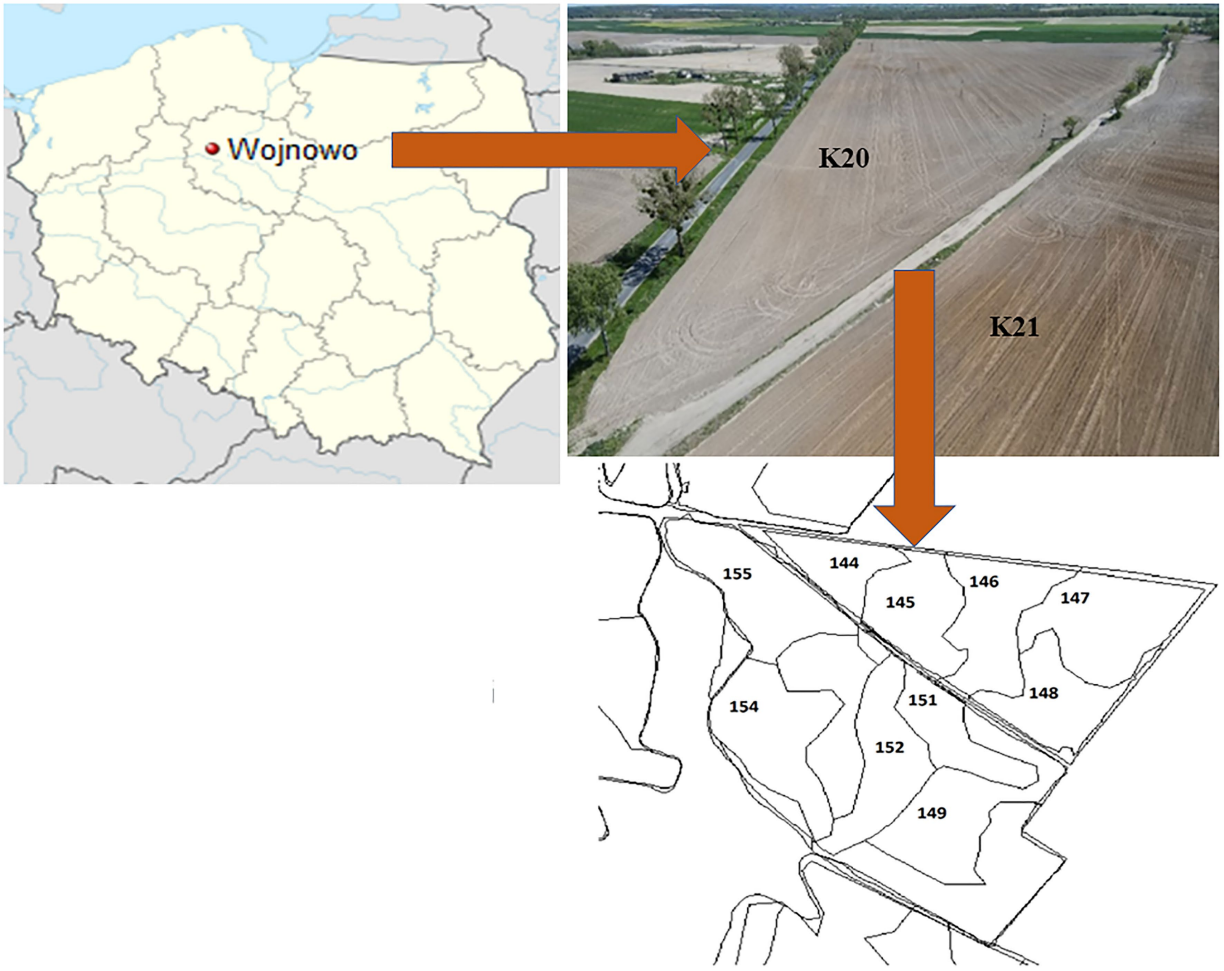
Study site location in Poland and view on the studied fields and established soil rasters.

The experimental site was located in Wierzchucin Królewski village (kujawsko-pomorskie voivodship, NW Poland) in the area belonging to the Potulicka Foundation Group headquartered in Wojnowo (Figure 1). The agricultural area held by the Potulicka Foundation is about 6,130 ha of farmland (about 4,980 ha of arable land and 1,150 ha of meadows). Over 60% of the crop in the Potulicka Foundation Group is maize grown for forage and grain. Other crops include wheat (approx. 20%), rapeseed (approx. 15%), and high-protein forage crops (lucerne, lupine; approx. 5%). The precision farming system is applied taking into account the differences in the nutrient abundance and irregular shapes of the fields (Figure 1). Importantly, more than 95% of the Potulicka Foundation cultivated area has been mapped using GPS. Thus, the management of crop production is carried out by means of a modern IT platform, which allows simultaneous data archiving and comprehensive statistical analysis of production processes. The arable land of the Potulicka Foundation consists of approximately 50% of class III and IV soils, while the other part of the area is located on class V and VI soils. Medium and light soils have a similar proportion, while heavy and very light soils constitute a small percentage of the area.

Two neighboring fields were selected for the experiments: K20 sown with a Gorzow mixture (intercropping mixture) to improve soil quality after a maize monoculture in 2020 and K21, where the long-term (over 30 years) monoculture cultivation was continued (Figure 1).

Field K20 (53.296°N, 17.790°E; Supplementary Figure S1) covers an area of 15 ha. This field was a perennial maize monoculture (over 30 years old). In 2020, it was sown with a Gorzow mixture (composed of perennial ryegrass, incarnate clover, and winter vetch) to improve the soil structure.

Field K21 (53.294°N, 17.788°E; Supplementary Figure S1) covers an area of 24 ha. It is adjacent to field K20 and is a perennial maize monoculture as well, except that it has never been sown with the intercropping mixture.

In each of the fields, five separate rasters were established (each about 3 ha in size), from which representative soil samples (0–20 cm) were taken (according to Polish Standard PN-ISO 10381-6; Polish Standard, 1998) three times a year: in spring (25.03.2020), summer (24.06.2020), and autumn (19.11.2020). In each raster, the soil samples were collected randomly (avoiding untypical soil areas) from approx. 20 to 30 sites to obtain representative soil material for each raster. Sampling was conducted in an automated manner using the IT system available on the Potulicka Foundation farms (Supplementary Figure S1), which allowed precise sampling from the same locations on each of these dates.

The soil of both fields was not fertilized before sampling in spring 2020 and in the autumn of 2019. However, fertilization was applied later (after soil sampling but before maize sowing) only in field K21 (K20 was not fertilized in 2020). In April 2020, potassium (K) fertilization was applied at a dose of 100 kg/ha of K salt with 60% K_2_O, as the current soil IT monitoring indicated a moderate abundance of K in the entire area of K21. Moreover, before maize sowing, nitrogen (N) fertilization in the form of urea 46% N was applied at a rate ranging from 150 through 175 to 200 kg/ha, depending on the yield potential in each raster of K21. During sowing (April 2020), localized/parallel fertilization (5 cm next to the seed and 5 cm below the sowing depth) was performed with ammonium phosphate (NP 18–46) at a fixed rate of 110 kg/ha (due to the moderate to high P content). The last liming treatment was carried out in the autumn of 2017 using magnesium lime variety 03 (Agrodol 03-RO from Omya) at doses from 1,000 to 4,000 kg/ha depending on pH in each raster.

In laboratory conditions, the soil samples were sieved through a 2-mm sieve and shortly stored in a refrigerator (4°C) until chemical analysis, whereas DNA extraction was performed immediately after sampling (no longer than 24 h after sampling).

### Soil Chemical Features

Soil acidity (pH) and redox potential (E_h_) were determined from a 2:1 soil suspension prepared in distilled water. An automatic multifunctional potential meter (Hach, Lange) equipped with glass and platinum measuring electrodes dedicated for pH and Eh determination, respectively, were used. All measurements were taken in triplicate after stabilization of the readings (Wolińska et al., 2017).

Total organic carbon (TOC) content was determined using an automatic carbon analyzer TOC-V_CSH_ SSM 5000A (Shimadzu, Japan). Soil samples (150 mg) were pulverized, dried prior to analysis, and then combusted at 900°C in a column containing a platinum and cobalt oxide catalyst (Wolińska et al., 2015). In these conditions, all carbon compounds were converted into the carbon dioxide form and detected by an infrared detector (Wolińska et al., 2015). All TOC recordings were realized in triplicate.

The soil moisture was determined with a gravimetric method (24 h, 105°C).

### DNA Isolation and Amplification

DNA extraction was performed in 0.350 g of soil within 24 h after sample collection using the commercial DNeasy PowerLyzer PowerSoil Kit (Qiagen, Germany) according to the manufacturers’ recommendations. Three independent replicates of DNA isolation were performed for each of the soil rasters. The quality and quantity of the DNA were analyzed in triplicate using a BioSpectrometer (Eppendorf, Hamburg, Germany). The fungal ITS region was amplified from each soil sample using the following primers (Schmidt et al., 2013) 5.8S (5'-GTC TCG TGG GCT CGG AGA TGT GTA TAA GAG ACA GCG CTG CGT TCT TCA TCG-3') and ITS1FI2 (5'-TCG TCG GCA GCG TCA GAT GTG TAT AAG AGA CAG GAA CCW GCG GAR GGA TCA-3'). For each sample, the PCR templates were adjusted to ~10 ng DNA and pooled in an equimolar concentration (Kuźniar et al., 2020). The PCR was performed using REDTaq® ReadyMix™ (Sigma, Saint Louis, Missouri, United States). The cycling conditions were 30 s at 98°C, followed by 25 cycles of 10 s at 98°C, 30 s at 55°C, and 20 s at 72°C, and a final elongation step of 2 min at 72°C. Library preparation was performed using the Q5 Hotstart High-Fidelity 2x Master Mix (New England Biolabs). Libraries were purified according to AMPure Beads XP (Beckman Coulter) manufacturer’s instructions. The next step was to index the samples with the Nexter XT Index Kit (Illumina) in a seven-cycle amplification reaction. The sequencing was performed by the company Genomed (PL).

### Next Generation Sequencing and Bioinformatic Analyses

The diversity of soil fungi was analyzed through amplicon sequencing on an Illumina MiSeq (Genomed S.A., Warsaw, Poland) using the paired-end (PE) technology with 2 × 300 nt with v3 chemistry according to manufacturer’s suggestions. Automatic analyses of the preliminary data were done using MiSeq Reporter (MSR) v2.6. They consisted of the following steps: trimming of adaptor sequences by the cutadapt program, quality control combined with trimming of low quality bases (quality < 20, min length – 30) with the cutadapt program, joining of paired reads with the use of the fastq-join algorithm, clustering with 97% sequence similarity with application of the UCLUST algorithm, detection and removal of chimeras by the usearch61 algorithm, and taxonomy assignment based on the UNITE v8 database by the blast algorithm.

Bioinformatic analyses were performed in R v4.1 using DADA2 v1.18 (Callahan et al., 2016), and sequences were classified using the DECIPHER package v2.20 (Wright, 2016) based on the reference database UNITE v2020_February2020 (Nilsson et al., 2019). The results are presented as the percentage of the relative abundance of sequences identified at the selected taxonomy levels (phyla, genera). Fungal biodiversity was also calculated using the diversity index (*H'*) according to Shannon-Wiener (Krebs, 1999) and the dominance (*D*) index according to Simpson (Krebs, 1999).

Statistical analyses were performed using the STATISTICA.PL package (10; Stat. Soft. Inc., Tulsa, OK, United States). The chemical data were subjected to ANOVA for comparison of means, and significant differences were calculated using the *post hoc* Tukey Honestly Significant Difference (HSD) test at *p* < 0.05 significance level (Wolińska et al., 2020).

## Results

### Chemical Soil Properties

Fluctuations in the basic chemical properties of the studied fields (K20 – intercropping mixture and K21 – maize monoculture) are summarized in Supplementary Table S1 and these results are influenced by the season (spring, summer, and autumn). In general, it was confirmed that precision farming maintained the correct pH of soils. A principle followed on the Potulicka Foundation farm is to apply liming on rasters, where the pH value falls below 6.0. The analyses of the changes in pH in the three terms of the vegetation season in both fields (Supplementary Table S1) showed the acidity range of 6.07–7.90 (K20) and 5.82–7.93 (K21) in spring, 5.70–6.63 (K20) and 5.31–6.60 (K21) in summer, and 5.99–6.94 (K20) and 5.84–6.99 (K21) in autumn. In the majority of the soil rasters, pH remained above 6.0 irrespective of the season, which is beneficial for both soil microorganisms and processes, and only single rasters with pH < 6.0 required liming (Supplementary Table S1).

The soil redox potential (E_h_) reached the highest values in spring (493.60–526.50 mV and 499.70–520.20 mV for K20 and K21, respectively) and summer (K20: 527.07–534.57 mV and K21: 537.73–568.03 mV), whereas slightly lower levels were recorded in autumn (K20: 417.73–499.13 mV and 426.17–515.39 mV). Nevertheless, in each term of the three seasons (Supplementary Table S1), E_h_ was maintained at a level favorable for the growth and development of soil microorganisms (>300 mV) and the recorded values confirmed that the studied soils were well-oxygenated (Szafranek-Nakonieczna and Stępniewska, 2015).

The soil organic carbon (TOC) content was low, but this is a typical characteristic of most Polish mineral soils. TOC ranged from 0.42 to 1.34% in the rasters of field K20 and from 0.23 to 1.02% in the K21 rasters (Supplementary Table S1). The dependence of the TOC concentration on the term of the vegetation season was also confirmed – the highest values were recorded in spring, while the lowest TOC was noted in autumn, which is directly related to the activity of metabolic processes carried out by microorganisms.

The soil moisture levels were fluctuated seasonally (Supplementary Table S1) by precipitation, which was most abundant in the summer and autumn of 2020, while the spring sampling was carried out after a snow-free winter. All these conditions were reflected in the results of the soil moisture level, which was lower in spring (K20: 4.59–8.92% and K21: 6.20–8.51%), moderate in summer (K20: 7.70–14.64% and 8.84–10.73%), and the highest in autumn (K20: 12.01–16.32% and K21: 9.61–13.35%).

### Evaluation of the Sequencing Data Quality

A summary of the sequencing data quality obtained in the current study is shown in Table 1. A total of 4,090,625 raw sequences were obtained (1,431,77, 750,676, and 1,908,173 for all samples taken in spring, summer, and autumn, respectively). In addition, 3,226,9,0 sequences remained after the preliminary quality filtering, which means that approx. 21% of poor quality sequences were removed during this stage. Then, denoised F/R quality filtering was done, which yielded 3,085,772 (denoised F) and 3,063,445 (denoised R) sequences for all of the three analyzed terms of the season. The total amount of merged forward-reverse reads was 2,637,055, while 2,632,578 sequences remained after chimera removal. This means that the performed steps resulted in the removal of approximately 35.6% of uncertain or poor quality sequences. Finally, the relative number of passed reads was in the range of 38–77%, depending on the analyzed raster (Table 1) with an average of about 66%.

**Table 1:** Sequencing data quality for the analyzed soil rasters during spring, summer, and autumn expressed by input – number of reads in raw fastq files; filtered – number of reads after preliminary quality filtering; denoised (F/R) – number of reads after quality filtering; merged – number of merged forward-reverse reads; nonchim – number of merged reads after removal of chimera sequences; and %passed – relative number of passed reads after all the above steps.

Number of raster	Input	Filtered	Denoised F	Denoised R	Merged	Nonchim	% Passed
**Spring**							
K20-144	127,745	102,019	98,558	98,419	86,870	86,803	68
K20-145	132,046	106,424	101,868	102,033	89,142	89,056	67
K20-146	131,436	106,621	101,664	101,177	86,716	86,646	66
K20-147	147,153	118,482	114,357	114,030	98,093	97,672	66
K20-148	185,209	156,578	153,548	153,309	140,808	140,469	76
K21-149	131,033	107,968	104,682	104,541	93,432	93,180	71
K21-151	129,029	99,820	95,737	94,143	83,119	82,294	64
K21-152	147,218	116,555	112,077	112,311	97,340	97,266	66
K21-154	189,573	139,379	136,018	136,225	123,920	123,770	65
K21-155	111,334	87,121	81,347	81,058	70,957	70,874	64
**Summer**							
K20-144	84,692	70,282	63,154	61,799	55,534	55,500	66
K20-145	91,288	76,905	71,716	70,587	62,001	61,953	68
K20-146	88,795	69,818	66,409	65,471	56,977	56,851	64
K20-147	68,166	52,584	48,058	47,263	36,145	36,110	53
K20-148	71,012	53,036	50,453	49,549	39,410	39,356	55
K21-149	137,096	119,501	116,669	116,577	105,319	105,169	77
K21-151	73,651	55,156	53,156	52,577	45,428	45,392	62
K21-152	5,121	33,469	30,271	29,297	19,709	19,705	38
K21-154	84,759	49,147	45,890	44,479	32,163	32,134	38
**Autumn**							
K20-144	200,759	159,635	150,244	150,499	125,878	125,656	63
K20-145	226,430	180,875	173,614	172,965	149,672	149,173	66
K20-146	233,657	192,772	181,205	174,897	154,944	154,721	66
K20-147	201,315	156,308	149,113	148,272	119,050	118,950	59
K20-148	130,977	92,641	87,921	87,737	71,036	70,966	54
K21-149	147,615	114,984	112,588	109,578	95,254	95,214	65
K21-151	190,379	150,684	142,219	142,190	119,435	118,863	62
K21-152	230,280	184,585	180,579	180,508	162,439	162,281	70
K21-154	168,259	131,411	126,447	126,038	106,047	105,882	63
K21-155	178,502	142,230	136,210	135,898	110,217	110,042	62

Rarefaction curves were also generated (Supplementary Figure S2) and plateaued for each sample (raster) studied, indicating good coverage and sequencing performance. The quality and quantity of DNA isolates are summarized in Supplementary Table S2.

### Fungal Biodiversity at the Phylum Level

**Figure 2:**
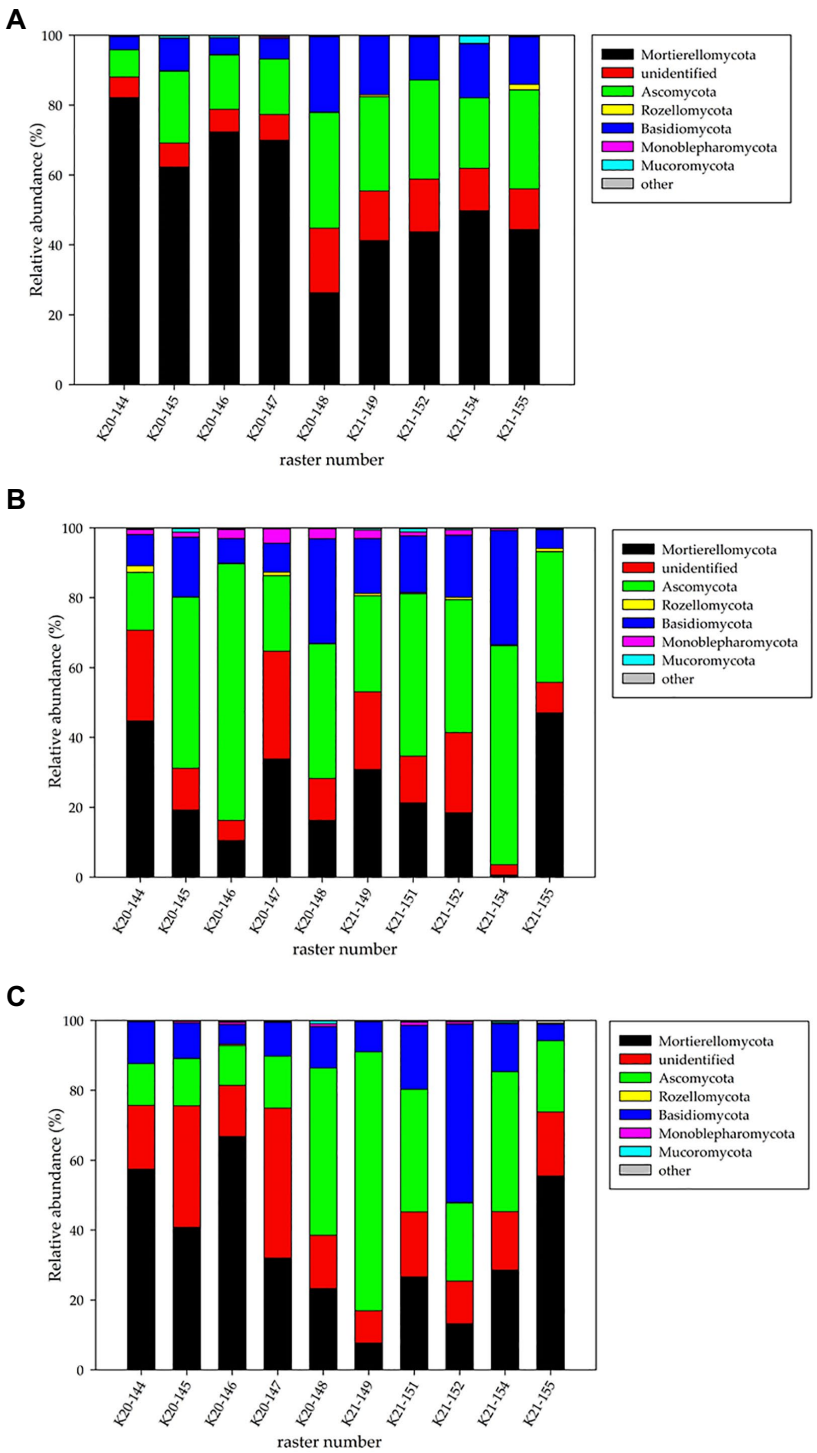
Relative abundance (%) of fungal phyla in the studied rasters of field K20 under the intercropping mixture and field K21 under the maize monoculture in spring **(A)**, summer **(B)**, and autumn **(C)** seasons.

The fungal phyla detected metagenomically in the studied rasters of field K20 under the intercropping mixture and field K21 under maize monoculture in spring, summer, and autumn are shown in Figure 2.

The findings indicated that fungal structure was affected by the cultivation system (intercropping mixture/maize monoculture) and the season. The NGS analyses identified six phyla of fungi (Mortierellomycota, Ascomycota, Basidiomycota, Monoblepharomycota, Rozellomycota, and Mucoromycota) in the fields studied (Figure 2); however, the dominance of the phyla changed depending on the season and cultivation system. The relative number of unidentified sequences at the phylum taxonomic level was approx. 2.9–3.1%.

In spring, Mortierellomycota dominated in both the fields, especially in field K20 (41.2–49.8% for K21 and 26.3–82.2% for K20). In summer, Ascomycota dominated in both fields (16.5–73.5% and 27.5–62.7% for K20 and K21, respectively), whereas Mortierellomycota were present as subdominants (K20: 10.5–42.7% and K21: 0.6–47%). A big difference was noticed in the structure of the fungal phyla recorded in autumn, when Mortierellomycota (23.2–66.8%) seemed to be the dominant type in field K20 and Ascomycota (20.4–74.1%) dominated in field K21, which clearly indicates an undeniable influence of the cultivation system on fungal biodiversity (Figure 2).

The third place in the phylum structure was occupied by representatives of Basidiomycota, whose relative abundance was higher in field K21 (12.3–51.1%) than in K20 (3.7–29.9%) in each of the three analysed seasons.

Representatives of the Rozellomycota, Monoblepharomycota, and Mucoromycota phyla were recorded with a substantially lower frequency in the rasters of both fields. For example, the presence of Rozellomycota in spring was confirmed in the K21-155 raster (Figure 2) at the level of 1.5% of identified sequences, but the appearance in summer was detected in five rasters in both fields K20 and K21 (0.7–1.9%). In turn, a very low level of these microorganisms (0.1–0.4%) was noted in autumn. Monoblepharomycota seemed to be the only phylum that was present only during the summer season with a higher number more higher than in the other two seasons. The presence of Mucoromycota was also demonstrated incidentally: in spring – rasters K21-154 (2.13%), K20-145, and K20-146 (0.57–0.64%), in summer – rasters K20-145 (1.21%), K20-149 (0.20%), and K21-151 (1.09%), and in autumn – rasters K20-148 (0.83%) and K21-154 (0.44%).

### Fungal Biodiversity at the Genus Level

The following criteria were used to classify the fungi at the genus level: dominant (accounting for >10% of identified sequences), subdominant (accounting for >2% of identified sequences), and accompanying (accounting for >1% of identified sequences), and the presence of these levels in at least one raster of the tested field was considered sufficient.

**Figure 3:**
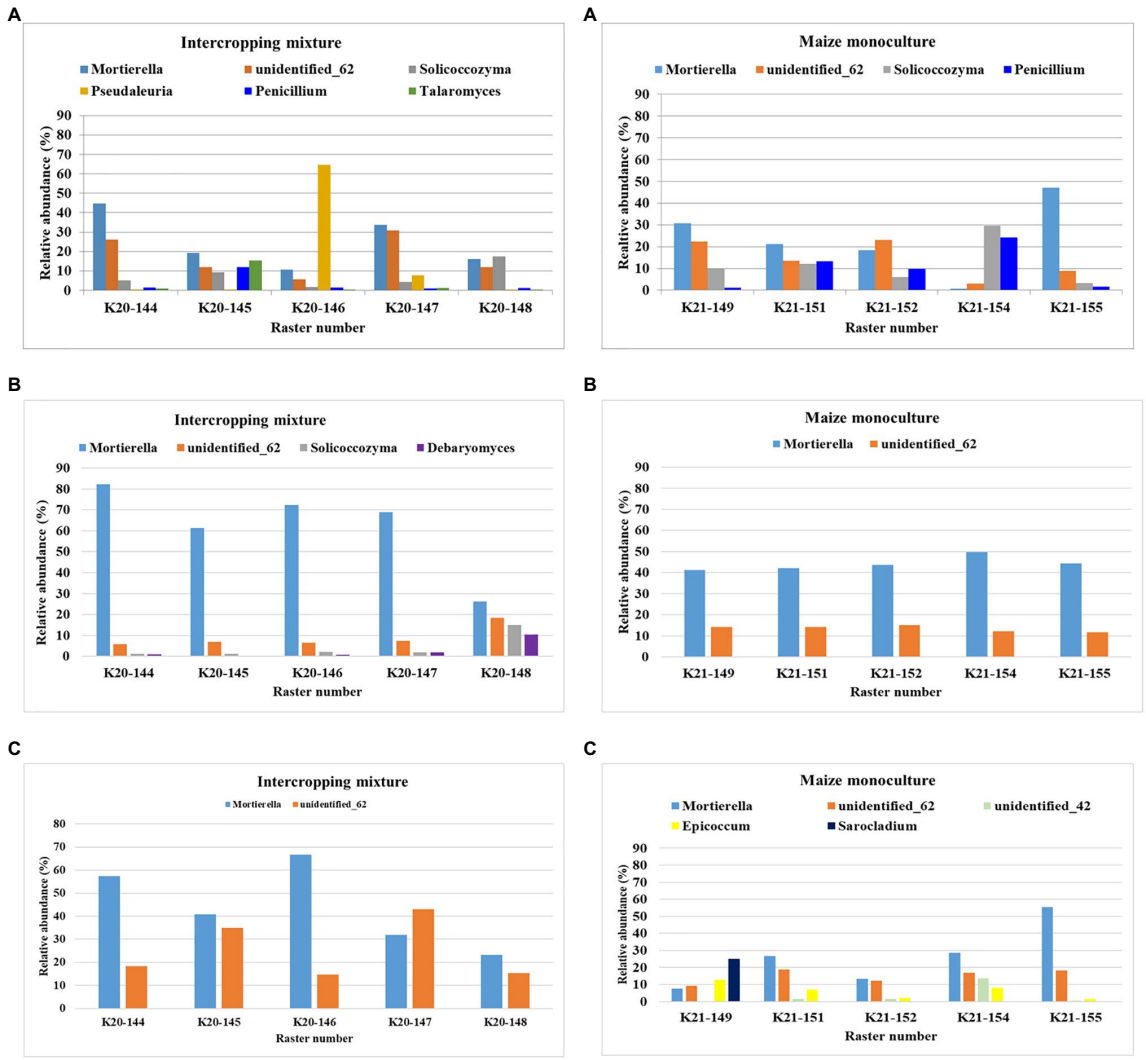
Dominant (>10%) fungal genera in the studied rasters of field K20 under the intercropping mixture and field K21 under the maize monoculture in spring **(A)**, summer **(B)**, and autumn **(C)** seasons.

The dominant (>10%) fungal genera in the rasters of field K20 under the intercropping mixture and field K21 under the maize monoculture in spring, summer, and autumn are presented in Figure 3. *Mortierella* was identified as the dominant genus in both fields; however, the relative abundance of these fungi was higher in field K20 than in K21. Seasonal variation in the dominant genus structure in the studied soils was confirmed as well. The highest abundance of *Mortierella* was recorded in summer in the K20 soil rasters (26–82% of identified sequences), followed by autumn (23–66.8%) and spring (10.5–44.7%). A similar trend was observed in field K21; however, the relative abundance of *Mortierella* was reduced by *ca*. 41% in summer and by 54–55% in autumn and spring compared to K20.

The second place in the structure of the dominant fungal genera was occupied by the genus *unidentified_62*, which was also subjected to seasonal fluctuations and depended on the mode of cultivation (Figure 3). This fungal genus was more abundant in the rasters of field K21 (maize monoculture), especially in summer (11.7–15.1%), than in K20 (intercropping mixture), where its relative abundance in summer amounted to 6.5% in the majority of the rasters and achieved the highest level in raster K20-148 (18.5%). However, this trend was reversed in autumn, when higher dominance of *unidentified_62* was demonstrated in field K20 (14.6–43%) than in K21 (9.3–18.3%).

The presence of *Solicoccozyma* was confirmed in both fields at the beginning of the vegetation season (in spring). It was more abundant in field K21 (3.2–29.7%) than K20 (1.7–17.5%). Already at the peak of vegetation (summer), the presence of this genus was confirmed only in field K20 (1.1–14.93%), whereas *Solicoccozyma* was not dominant in autumn (Figure 3).

*Penicillium* as the dominant genus (11.99%) was found only in spring in one raster (K20-144) and its higher frequency was noted in field K21 (9.8–24.12%). *Talaromyces* was present as the dominant genus in spring samples taken from the K20-145 raster (10.49%), while the presence of *Debaryomyces* was confirmed in the K20-148 raster (10.35%) during the summer sampling time (Figure 3). Interestingly, K21 was characterized by higher biodiversity of dominant genera in individual rasters in autumn compared to K20. The following fungal genera were found in field K21 in autumn: *unidentified_42* (K21-144: 13.8%), *Epicoccum* (K21-149: 12.7%), and *Sarocladium* (K21-149: 25.03%).

The variability in the subdominant (>2%) and accompanying (>1%) fungal genera as an effect of the season and the mode of land use (K20 and K21) is shown in Supplementary Figures S2, S3, respectively. The following genera were identified as subdominants in this study: *Exophiala*, *Podosphora*, *Pseudaleuria*, *Fusicolla*, *Oidodendron*, *Peziza*, *Pseudogymnoascus*, *Humicola*, *Trichoderma*, *Sporobolomyces*, *Nadsonia*, *Fusarium*, and *Metarhizum*, and some unidentified genera marked as *unidentified_88*, *unidentified_9826*, *unidentified_5*, *unidentified_12*, *unidentified_59*, *unidentified_1560*, *unidentified_42*, and *unidentified_364* (Supplementary Figure S2). A tendency was noticed toward the persistence of a relatively higher abundance of subdominant fungal genera in field K21 than in K20, which suggests that skillfully implemented principles of precision agriculture contribute to the preservation of biodiversity even in perennial maize monocultures (Supplementary Figure S2).

Accompanying (>1%) fungal genera were represented by fungi of *Pseudeurotium*, *Chaetomium*, *Fusicolla*, *Cladophialophora*, *Umbelopsis*, *Apodus*, *Candida*, *Rhodotorula*, *Malassezia*, *Epicoccum*, and unidentified genera referred to as *unidentified_73*, *unidentified_7*, and *unidentified_5* (Supplementary Figure S3). Both in spring and in autumn, the relative abundance of accompanying fungal genera in field K21 was limited in comparison with that in field K20.

### Fungal Indicators of Sensitivity to Maize Monoculture

**Figure 4:**
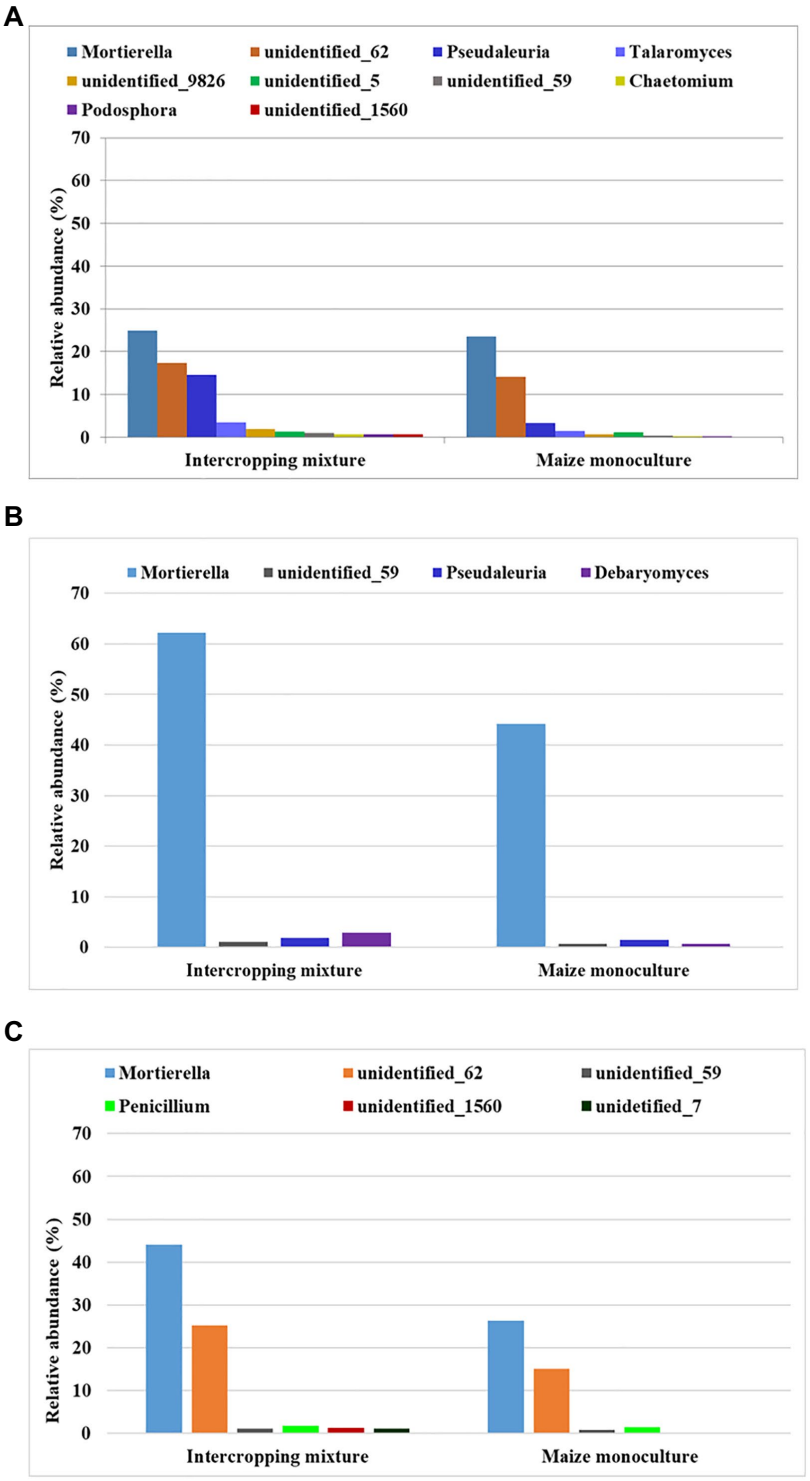
Fungal genera displaying sensitivity to maize monoculture during spring **(A)**, summer **(B)**, or autumn **(C)** seasons.

Among the three groups (dominant, subdominant, and accompanying) of fungal genera, we selected those that displayed sensitivity to maize monoculture indicated by their relative abundance (Figure 4). In other words, we distinguished genera that responded by decreasing their relative abundance in field K21 compared to K20.

Such a relationship was represented by as many as 10 genera of fungi in spring (*Mortierella*, *Pseudaleuria*, *Talaromyces*, *Chaetomium*, *Podosphora*, and five representatives of unidentified genera), only four genera in summer (*Mortierella*, *Pseudaleuria*, *Debaryomyces*, and *unidentified_59*), and six genera in autumn (*Mortierella*, *Penicillium*, and four unidentified genera; Figure 4).

**Figure 5:**
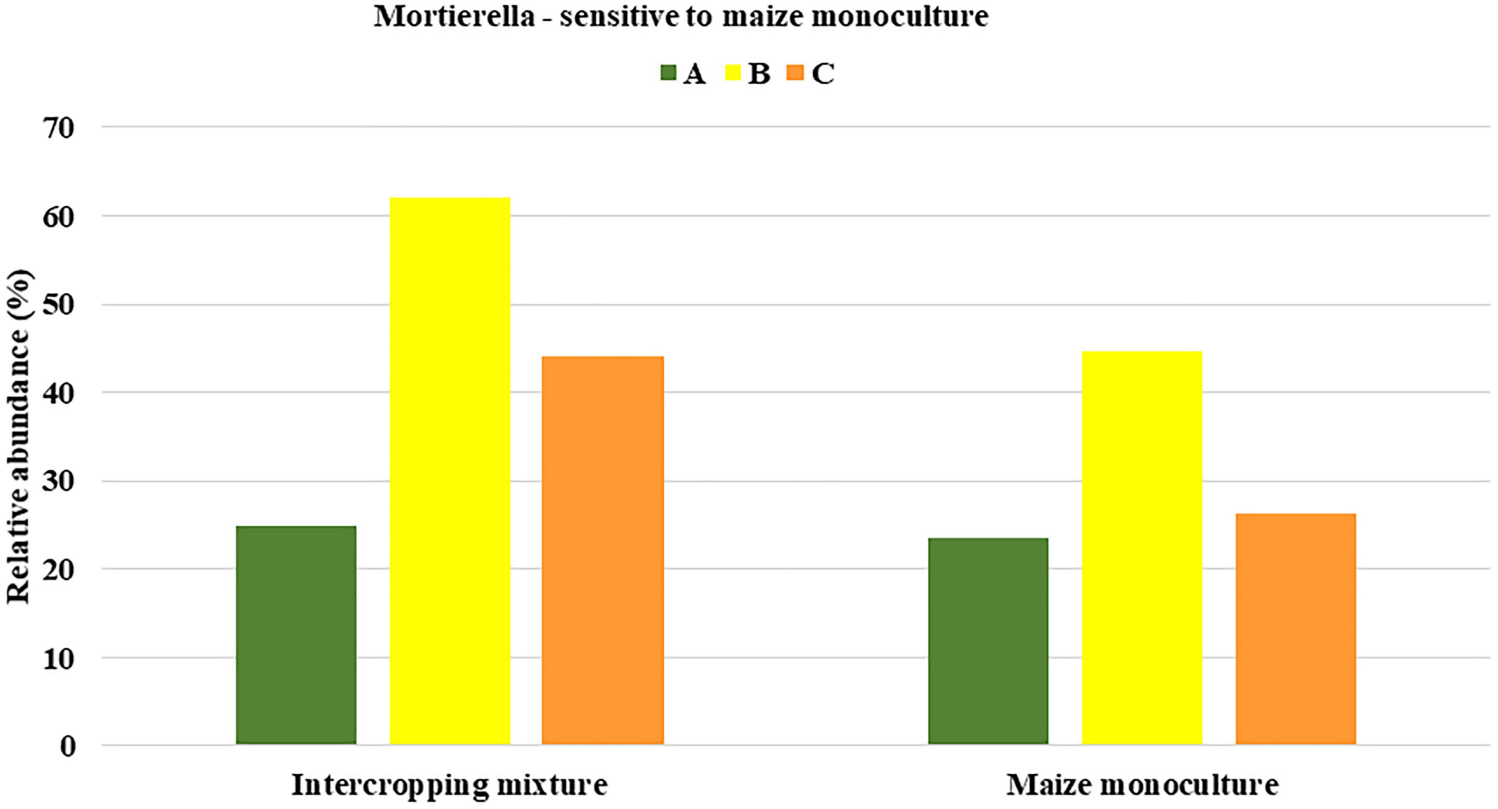
Fungal genus recommended as sensitive to maize monoculture during spring (A), summer (B), or autumn (C) seasons.

To be sure that the recommendation of a fungal indicator of sensitivity to long-term monoculture crops could be universal enough to show the predicted trend in the changes regardless of the date in the vegetation cycle, it was assumed that a decrease in the relative abundance of this indicator should occur in each of the three analyzed seasons. Consequently, only one of the identified genera, *Mortierella*, met such a requirement (Figure 5). It was evidenced that the relative abundance of *Mortierella* was higher in the soil under the intercropping mixture than in the soil under the long-term maize monoculture and this dependence was maintained regardless of the sampling term. More specifically, it was reported that the relative abundance of *Mortierella* in field K21 decreased by 5% compared to field K20 in spring. The decrease amounted to 28% in summer and as much as 40% in autumn (Figure 5).

### Fungal Indicators of Resistance to Maize Monoculture

**Figure 6:**
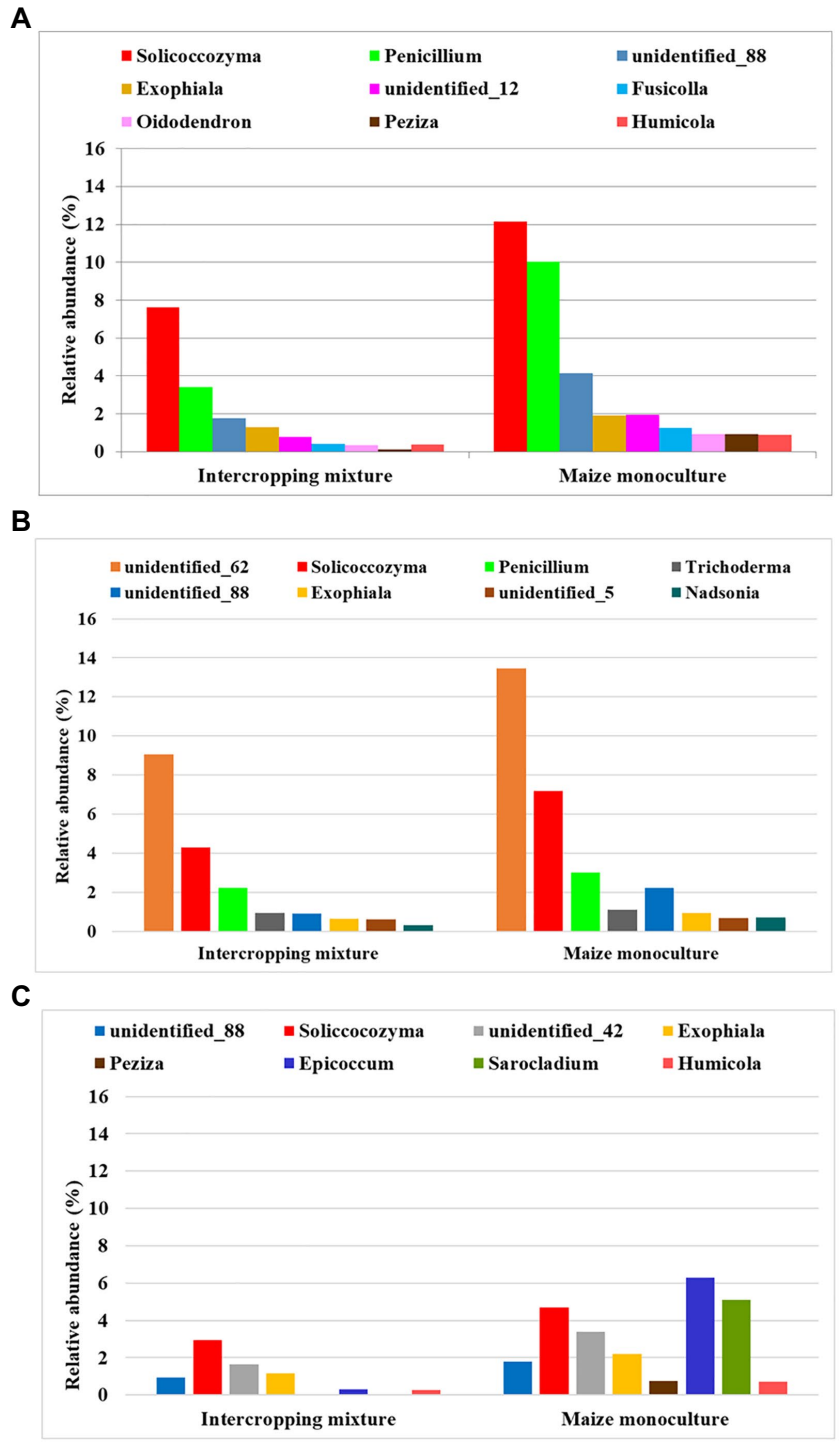
Fungal genera displaying resistance to maize monoculture during spring **(A)**, summer **(B)**, or autumn **(C)** seasons.

An analogous principle to that described in the previous subsection was adopted in the search for indicators of fungi that prefer monoculture soils for colonization and show resistance to the lack of crop rotation. Consequently, we distinguished genera that responded by increasing their relative abundance in field K21 (maize monoculture) compared to field K20 (intercropping mixture), and this dependence was illustrated in Figure 6.

Such a trend was demonstrated by nine fungal genera (*Solicoccozyma*, *Penicillium*, *Exophiala*, *Fusicolla*, *Oidodendron*, *Peziza*, *Humicola*, and two unidentified representatives) in spring. In summer, eight genera reacted by increasing their relative abundance in field K21 (*Solicoccozyma*, *Penicilium*, *Trichoderma*, *Exophiala*, *Nadsonia*, and three unidentified genera), and genera were included in this group in autumn (*Solicoccozyma*, *Exophiala*, *Peziza*, *Epicoccum*, *Sarocladium*, *Humicola*, and two unidentified genera).

**Figure 7:**
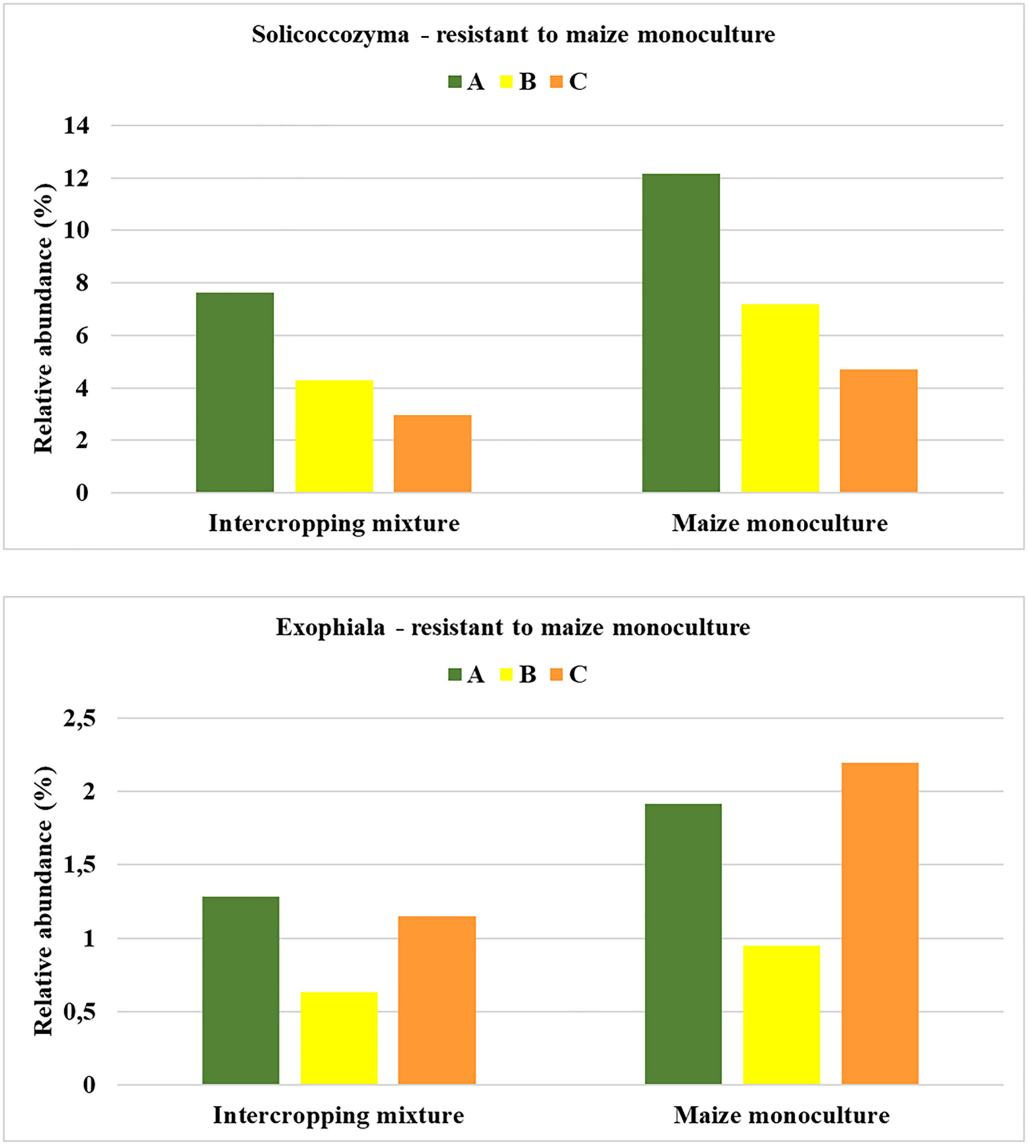
Fungal genera recommended as resistant to maize monoculture during spring (A), summer (B), or autumn (C) seasons.

To be sure that the recommendation of a fungal indicator of resistance to long-term monoculture crops could be universal enough to show the predicted trend in the changes regardless of the date in the vegetation cycle, it was assumed that an increase in the relative abundance of this indicator should occur in each of the three analyzed seasons. Such rigorous criteria were fulfilled by two fungal genera: *Solicoccozyma* and *Exophiala* (Figure 7). It was evidenced that the relative abundance of *Solicoccozyma* in spring increased by 59% in field K21 compared to field K20. The increase was 67% higher in summer and 58% higher in autumn than in the intercropping mixture field (Figure 7). A similar trend was demonstrated by *Exophiala* representatives, as their relative abundance in field K21 was 49% higher in spring and summer and up to 90% higher in autumn in comparison with field K20. This indicates that both genera are adequate indicators of resistance to long-term maize monocultures and can be regarded as resistance indicators.

### Beta-Diversity and Biodiversity Indices

**Figure 8:**
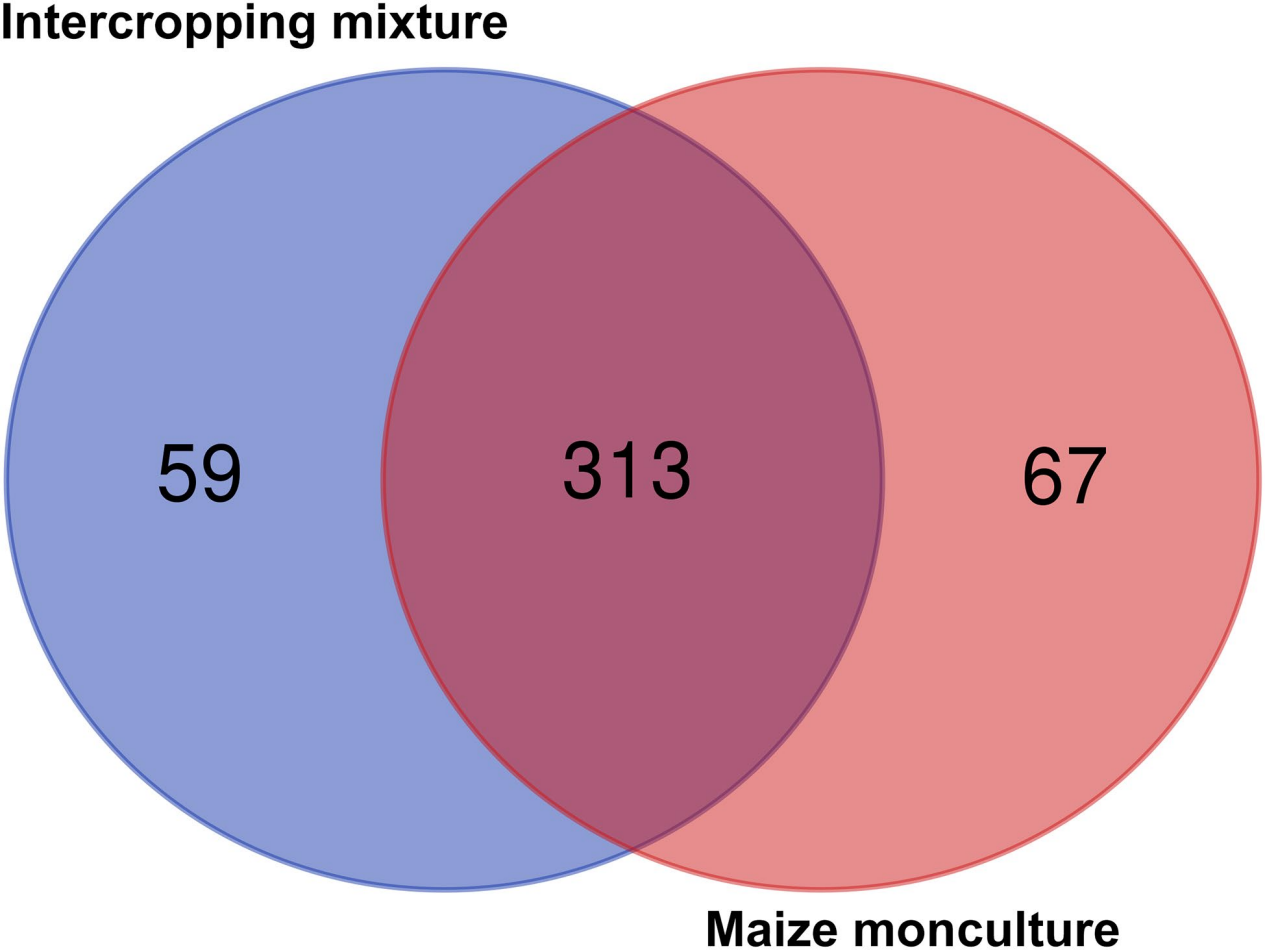
Venn diagrams illustrating numbers of common and different fungal genera in soils under the intercropping mixture and the maize monoculture.

Venn diagrams illustrating the relative abundance of shared and unique fungal genera in soils under the intercropping mixture and maize monoculture are shown in Figure 8. In total, 356 fungal genera were identified in the current experiment, of which 50% (178 genera) appeared to be common genera present in both fields, whereas a higher abundance of differential genera (125) was noted in field K21 (the maize monoculture) than in field K20 (53) sown with the intercropping mixture (Figure 8). This trend may be connected with the fact of fertilization of field K21 during the 2020 vegetation season.

**Figure 9:**
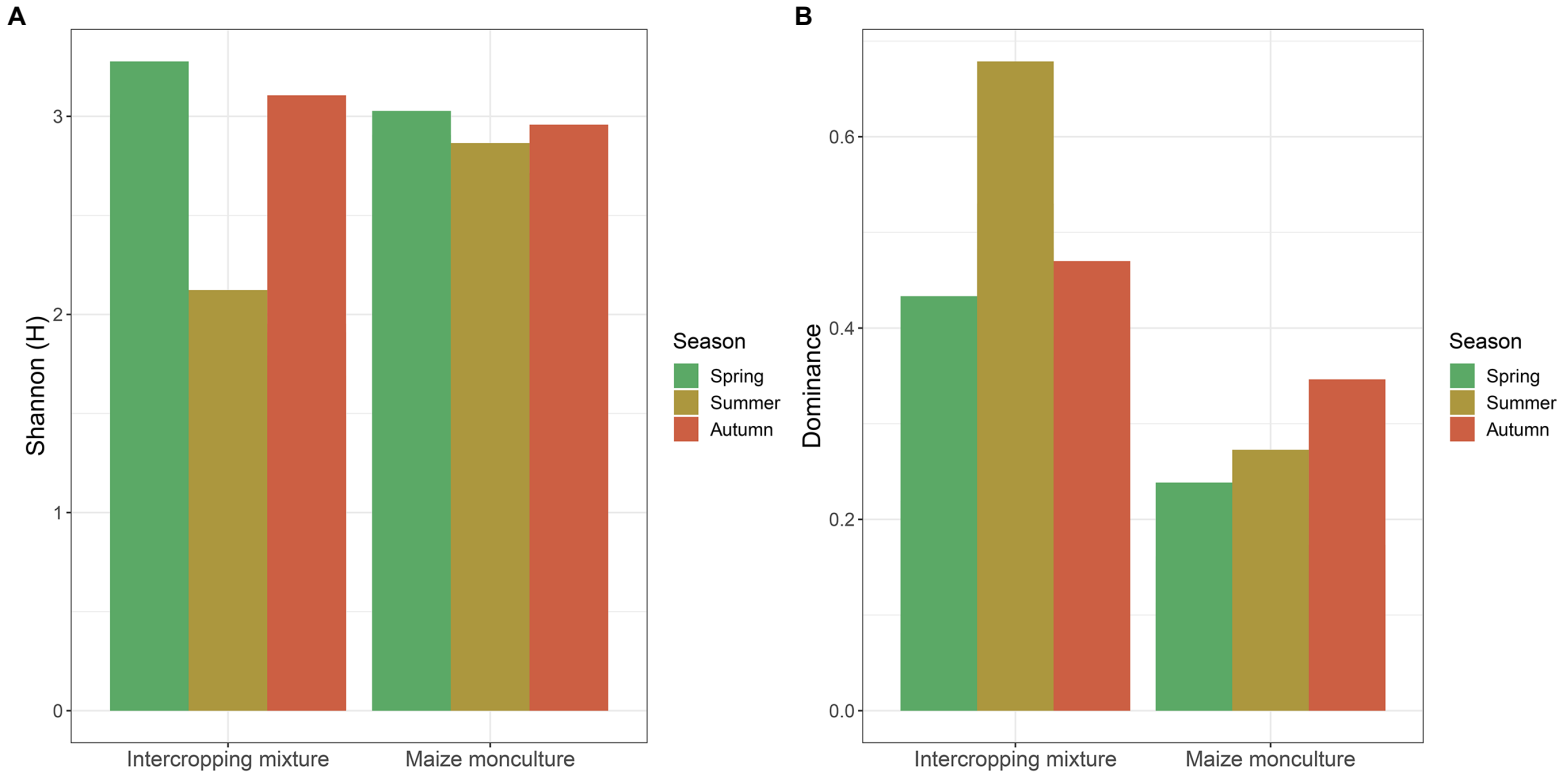
**(A)** Shannon-Weaver (*H'*) biodiversity and **(B)** Simpson dominance (*D*) indices values at different terms of the vegetation season in the fields under the intercropping mixture and the maize monoculture.

Fungal biodiversity expressed by Shannon-Wiener (*H'*) and Simpson dominance (*D*) indices at the different terms of the vegetation season in the fields under the intercropping mixture and maize monoculture is presented in Figure 9. Generally, the analysis of the *H'* values indicates that the biodiversity at the beginning of the vegetation season (spring) was higher in the intercropping mixture field (*H'* 3.34) than in the maize monoculture site (*H'* 3.01). The situation changed in summer, when the fungal biodiversity increased in the monoculture field (*H'* 2.89) compared to the neighboring K20 (*H'* 2.09) in response to the fertilization of field K21 (Figure 9). However, at the end of the growing season after maize harvest (autumn), similar levels of biodiversity ranging from *H'* 2.97 (K21) to *H'* 3.11 (K20) were recorded in both fields.

The values of the dominance (*D*) index confirmed the higher biodiversity in the maize monoculture field (0.22–0.35) than in the intercropping mixture variant (0.43–0.68) in each of the three terms (Figure 9), which confirmed the effect of fertilization applied in field K21 and the absence of fertilization in field K20.

## Discussion

Biodiversity loss is one of the most serious global environmental problems caused by human activities (Fuchs et al., 2021). Fungi in the soil play the role of saprophytes, reducers, and symbionts and have an important function in the generation of crop yields (Klaubauf et al., 2010; Jezierska-Tys et al., 2020). Therefore, knowledge of their presence and structure in agricultural soils is desirable. Moreover, there is little information about soil fungal communities in diverse locations with distinct soil types (Chen et al., 2016). Hence, there is a great need for detailed investigations of the structure of soil fungi under long-term fertilization (Ullah et al., 2019) and long-term monocultures.

The NGS analysis facilitated precise recognition of the actual state of the mycobiome in two fields: one sown with an intercropping mixture and the other under maize monoculture. We evidence that the mycobiome biodiversity is dependent on both the cultivation system (intercropping mixture/maize monoculture) and the season (spring, summer, and autumn). This confirms the results reported by He et al. (2017), Sommermann et al. (2018), and Jezierska-Tys et al. (2020), which suggested that both cultivation systems and vegetation phenology may be responsible for the differentiated seasonal variations of fungal diversity. Li et al. (2019), indicate that the fungal richness and diversity in acidic red forest soils in summer and autumn are higher than in spring. We also prove that even a 1-year break in the monoculture in favor of the intercropping mixture has a positive effect and improves the soil properties. It is found that the application of the intercropping mixture compared with the maize monoculture (Supplementary Table S1) results in an increase in the soil moisture by about 8.5% in spring, 3.2% in summer, and 16% in autumn. The TOC content is increased by approximately 37% in spring, 26% in summer, and 71% in autumn. Both factors (moisture and organic carbon) are crucial for the growth and presence of soil microorganisms in the soil environment (Choudhary et al., 2018; Frąc et al., 2018; Bojarszczuk et al., 2019; Liddle et al., 2020). As reported by Semchenko et al. (2018), Grządziel and Gałązka (2019), and Adamo et al. (2021), pH has a decisive influence on soil fungal diversity. Importantly, pH in the studied soils is perfectly regulated by the rules of precision agriculture and, consequently, is at the level of approx. 6.0 (Supplementary Table S1). However, it should be emphasized that plant (e.g., maize) cultivation in permanent monoculture is in general connected with one-sided exhaustion of nutrients and, consequently, changes in the biodiversity structure (Wang et al., 2011; Furtak et al., 2017; Gałązka et al., 2017; Liang et al., 2011; Gałązka and Grządziel, 2018; Bojarszczuk et al., 2019). Urbanova et al. (2015) report that forest fungi may respond to soil nutrients such as phosphorus (P) or nitrogen (N) and that trees may affect ecosystem properties through several processes, including production of litter or *via* root exudates. Burke et al. (2019) suggests that microsite chemical factors (soil moisture, P availability) correlate more strongly with fungal community variation than climate factors (soil temperature).

Moreover, planting the same crop every year in the same field promotes the spread of adapted weeds, pathogens, and other pests (Moritz et al., 2021). All this makes monoculture soils a difficult habitat for microbial colonization, and they are regarded as ecological niches with a rather low biodiversity and poor fertility (Bojarszczuk et al., 2019).

Among the fungal phyla identified in the current study, representatives of Mortierellomycota, Ascomycota, and Basidiomycota dominate, whereas Rozellomycota, Monoblepharomycota, and Mucoromycota are accompanying phyla represented with rather low frequency (Figure 2). In soils under maize monoculture, Gałązka and Grządziel (2018) note Zygomycota, Basidiomycota, and Ascomycota as the main phyla. Frąc et al. (2021) studied changes in soil fungal diversity as an effect of spent mushroom substrate and chicken manure treatment and found Ascomycota, Basidiomycota, and Mortierellomycota phyla as dominants in the fungal structure. In addition, Chytridiomycota, Mucoromycota, Rozellomycota, and Zoopagomycota are identified as minor phyla (Frąc et al., 2021). Grządziel and Gałązka (2019) confirm the dominance of Ascomycota and Basidiomycota in the majority of Polish agricultural soil types, while Mortierellomycota is in the third place in the mycobiome structure. A similar distribution of fungal phyla is confirmed by Klaubauf et al. (2010) in agricultural soils from Lower Austria. In general, our results are in agreement with the reports indicated above, and the only difference can be found in the percentage abundance of each phylum in the fungal mycobiome structure. In the current study, Mortierellomycota seems to be the most numerous phylum, irrespective of the season of the year (Figure 2), which is beneficial from an ecological point of view. Fungi belonging to this phylum are commonly found in various environments, e.g., bulk soil, rhizosphere, and plants (Frąc et al., 2021), and are known as plant growth-promoting fungi (Ozimek and Hanaka, 2021). It should be noted that representatives of Mortierellomycota fungi live as saprotrophs in the soil, on decaying leaves, and on other organic materials (Frąc et al., 2021; Ozimek and Hanaka, 2021). Moreover, they have the ability to decompose chitin and hemicellulose to obtain the sugars needed for their growth and development (Frąc et al., 2021; Ozimek and Hanaka, 2021). Ascomycota is characterized by high abundance as well; thus, it can be concluded that this phylum adapts well to the conditions of long-term maize monocultures. Basidiomycota demonstrated similar properties, as they were relatively more abundant in the rasters of field K21 compared to field K20. It is known that Basidiomycota includes some of the most familiar fungi that cause wood decay, decompose litter effectively, and improve the storage of water, metabolites, and minerals (Millanes et al., 2011; Frąc et al., 2021). Rozellomycota is also noteworthy, although it occurred at a lower frequency in the studied soils (Figure 2). This taxon of microorganisms is classified either as fungi or as a sister group of fungi; it differs from classical fungi in that they lack chitinous cell walls at every trophic stage of the life cycle. Hence, they are described as phagotrophic parasites that feed by attaching to, absorbing, or living inside other cells. Grządziel and Gałązka (2019) demonstrated that some of the fungi belonging to Rozellomycota prefer soils with extreme (acidic) pH values. This fact may explain the low percentage of this taxon in the structure of the monoculture soils studied in the current paper, as the pH of these soils was regulated and kept above the 6.0 limit in most rasters (Supplementary Table S1).

At the genus level, we found that *Mortierella* is the dominant genus in both fields; however, its relative abundance is higher in field K20 than in K21 (Figure 3). It should be underlined that *Mortierella* representatives are common fungi establishing mutualistic relationships with other fungi or bacteria (Ozimek et al., 2018; Zhang et al., 2020; Ozimek and Hanaka, 2021). *Mortierella* species are classified as saprotrophic microorganisms isolated from forest litter; recently, their status as highly valuable organisms in agricultural soils has been confirmed (Ozimek et al., 2018; Frąc et al., 2021; Ozimek and Hanaka, 2021). Such key characteristics as the ability to survive in very unfavorable environmental conditions (e.g., monoculture soils, as demonstrated by the present study) and the utilization of carbon sources contained in cellulose, hemicellulose, and chitin polymers make these fungi effective agricultural inoculants (Ozimek et al., 2018; Ozimek and Hanaka, 2021). Therefore, their presence in long-term maize monocultural soils is favorable from the ecological point of view, especially since it is suggested that the activities of *Mortierella* species selected from cultivated plants influence the soil microbiota and support the performance of beneficial microorganisms enhancing crop yields substantially (Zhang et al., 2020; Ozimek and Hanaka, 2021). Representatives of the genus *Solicoccozyma* are identified as well among the genera of fungi dominating in the investigated soils (Figure 3). The dominance of both *Mortierella* and *Solicoccozyma* has been confirmed by Frąc et al. (2021) in soils under long-term application of spent mushroom substrate and chicken manure. Similarly, Grządziel and Gałązka (2019) have observed the presence of the *Mortierella* and *Solicoccozyma* genera irrespective of pH and agricultural soil type. However, no *Solicoccozyma* representatives are detected in soil under long-term monoculture of maize cultivated with various techniques (Gałązka and Grządziel, 2018), which differentiates these results from the findings of the present study.

As reported by other authors, *Aspergillus*, *Penicillium*, *Fusarium*, *Trichoderma*, *Rhizopus*, *Mucor*, *Cladosporium*, *Verticillium*, *Acremonium*, *Chaetomium*, *Eurotium*, and *Corynespora* are comparatively more frequent than other species in most soil samples (Jayaraman et al., 2018). Sudarma et al. (2011) have identified *Streptomyces*, *Trichoderma*, *Aspergillus*, *Penicillium*, and *Gliocladium* as representative inhabitants of banana fields from Bali – the main banana growing areas in the world, whereas the most common genera isolated from Indian agricultural soils included *Penicillium*, *Aspergillus*, *Acremonium*, *Fusarium*, *Mortierella*, *Mucor*, *Paecilomyces*, *Talaromyces*, *Trichoderma*, and *Verticillium* (Swer et al., 2011). In soil under long-term maize monoculture, Gałązka and Grządziel (2018) have confirmed the presence of the following fungal genera: *Penicillium*, *Cladosporium*, *Verticillium*, *Epicoccum*, *Geomyces*, *Mucor*, *Cryptococcus*, *Hypocrea*, *Pseudogymnoascus*, *Preussia*, and *Plectosphaerella*. In general, our results are in agreement with the aforementioned findings, as some of the fungal genera are also identified in the current study as the dominant (Figure 3), subdominant (Supplementary Figure S3), or accompanying (Supplementary Figure S4) mycobiome of maize monocultural soils. However, in the studied fields, we also note the presence of *Exophiala*, *Podoshpra*, *Pseudoleuria*, *Fusicolla*, *Oidodendron*, *Peziza*, *Sporobolomyces*, *Nadsonia*, *Metharizum*, *Humicola* (Supplementary Figure S3), *Cladophialophora*, *Umbelopsis*, *Apodus*, *Candida*, *Rhodototula*, and *Malassezia* (Supplementary Figure S4). This confirms the diverse biodiversity of monoculture soils and no degradation of fungal biodiversity by precision agriculture principles.

There is no doubt that microbial diversity is an important ecological indicator and it is generally believed that the higher the microbial diversity in the soil, the more stable the ecosystem (Chaer et al., 2009). Hence, the microbial community structure and abundance are regarded as vital indicators of soil quality (Ullah et al., 2019). In the current study, the fungal biodiversity is also reflected by the determined values of biodiversity indices (Figure 9), which confirmed its variability depending on both the cultivation mode and the season. As indicated by the *H'* index, the highest biodiversity was noted in field K20 (intercropping mixture) in spring and autumn. The dominance index suggests that field K20 is characterized by higher biodiversity of the dominant fungal genera, while field K21 contains lower numbers of dominant genera in the fungal mycobiome structure, which indicates *de facto* higher diversity represented by a higher number of less abundant genera (Figure 9). This trend is also confirmed by the Venn diagram (Figure 8). Similarly, as reported by Gałązka and Grządziel (2018), the sampling time and cultivation technique have a great influence on the fungal community structure. The highest *H'* index is calculated (Gałązka and Grządziel, 2018) for soil taken before maize sowing (in spring), as in the current study.

The undoubted novelty of the current work is the recommendation of fungal indicators of both sensitivity and resistance to long-term maize monoculture cultivation. To the best of our knowledge, there is no similar work in the available literature. Based on obtained results, we recommend *Mortierella* as a potential indicator of sensitivity to long-term maize cultivation (Figure 5), whereas *Solicoccozyma* and *Exophiala* can be proposed as indicators of resistance to long-term maize cultivation (Figure 7).

The important ecological role of the presence of *Mortierella* in the soil environment is discussed above, whereas the role and function of *Solicoccozyma* in agricultural soils mainly involves biodegradation features (Stosiek et al., 2019), i.e., the split of phosphorus to nitrogen and nitrogen to carbon bonds in N-phosphono-methylglycine (PMG, glyphosate). *Solicoccozyma* is also known for its production of indole-3-acetic acid (IAA), which is the most common phytohormone occurring in plants and regulating various aspects of plant growth and development (Nicola et al., 2021). Consequently, the presence of these fungal genera in maize monoculture soils is desirable and important from the ecological point of view.

*Exophiala* species are common environmental fungi often associated with decaying wood and known as a source of biologically active metabolites with cytotoxic activity (Wang et al., 2011). Moreover, several *Exophiala* species have been reported to be able to degrade benzene, toluene, ethylbenzene, and xylenes (Zhang et al., 2019). As reported by Macia-Vicente et al. (2016), *Exophiala* constitutes a polymorphic group of ascomycetous fungi in the family Herpotrichiellaceae (Chaetothyriales). It includes dematiaceous anamorphic species characterized by annellidic conidiogenesis and frequent yeast-like states (Macia-Vicente et al., 2016). Some studies of *Exophiala* representatives have investigated their importance as etiologic agents of diseases in animals and humans (Najafzadeh et al., 2013; Wen et al., 2016). However, the pathogenic lifestyle of *Exophiala* species is rather opportunistic, and members of the genus are frequently isolated from natural environments, e.g., bulk soil, rhizosphere, rock surfaces, air, water, and plant tissues (De Hoog et al., 2011; Ferrari et al., 2011).

The response of the aforementioned fungal genera to long-term maize monoculture by either increasing or decreasing their incidence irrespective of the stage of the maize growing cycle observed in the current work is their new feature and has not been reported in the literature to date. Furthermore, we believe that the introduction of basic biotic indicators to soil analysis may be future-oriented. However, it should be emphasized that the costs of introducing ours indicators into basic microbiological soil analysis are still high for the average farmer. We also trust that our research on soil microbiological monitoring can contribute to the development of an artificial network algorithm that learns to predict soil microbiological aspects based on the analysis of abiotic factors.

## Conclusion

This study was conducted using high-throughput sequencing to investigate the diversity and composition of soil fungal communities and their seasonal variations in two agricultural fields under long-term maize cultivation. In one of them, an intercropping mixture was sown after a monoculture of maize in 2020 to improve soil quality. In turn, the long-term (over 30 years) monoculture cultivation was continued in the other field. The NGS analysis proved that the fungal structure was dependent on both the seasons and the agricultural practices. Our results evidenced that even a 1-year break in the monoculture in favor of the intercropping mixture had a positive effect and improved soil properties promoting the occurrence of higher biodiversity (moisture, pH, and TOC). Among the fungal phyla, representatives of Mortierellomycota, Ascomycota, and Basidiomycota were found as dominants, whereas Rozellomycota, Monoblepharomycota, and Mucoromycota occurred as accompanying phyla. At the genus level, *Mortierella* was identified as a dominant genus in both fields. However, the relative abundance of these fungi was higher in the field under the intercropping mixture rather than in the field under the long-term continuous maize cultivation, and this was observed regardless of the season of the year. Therefore, *Mortierella* was recommended as a fungal indicator of sensitivity to maize monoculture. By contrast, *Solicoccozyma* and *Exophiala*, which regardless of the season responded by increasing their abundance in soil under the long-term monoculture in comparison to the intercropping mixture variant, were recommended as indicators of resistance to continuous maize cultivation (without crop rotation). Finally, it should be emphasized that the precision farming principles applied on the Potulicka Foundation farm have a very positive effect, as evidenced by the fungal biodiversity, which is differentiated even in the long-term maize monoculture field. Therefore, monoculture cultivation carried out in this way does not induce biological degradation of this type of soil when both pH and organic carbon content are systematically controlled.

Further research is recommended to determine both fungal and bacterial indicators for monoculture crops based on observations of more than one vegetation season to formulate comprehensive conclusions. Another important research perspective is to include other crops, such as wheat and rapeseed, in the study of bacterial and fungal indicators. Finally, it is also worth conducing further studies on the effect of chemical and biological soil features on fungal indicators, which will be helpful in understanding the impact of different factors on the fungal structure in agricultural soils.

## Data Availability Statement

The datasets presented in this study can be found in online repositories. The names of the repository/repositories and accession number(s) can be found at: https://www.ncbi.nlm.nih.gov/, PRJNA725644.

## Author Contributions

AW designed the research, wrote the manuscript, and has responsibility for the final content. AK, AG, and JG performed the laboratory analyses and prepared the figures. JP and AS prepared the experimental fields and sampled the soil material. All authors interpreted the results. All authors contributed to the article and approved the submitted version.

## Funding

The study was supported by the Potulicka Foundation Economic Center (in the frame of UKDKW 2020/03/1).

## Conflict of Interest

The authors declare that the research was conducted in the absence of any commercial or financial relationships that could be construed as a potential conflict of interest.

## Publisher’s Note

All claims expressed in this article are solely those of the authors and do not necessarily represent those of their affiliated organizations, or those of the publisher, the editors and the reviewers. Any product that may be evaluated in this article, or claim that may be made by its manufacturer, is not guaranteed or endorsed by the publisher.
