# Supplementary material for: Fungal Indicators of Sensitivity and Resistance to Long-Term Maize Monoculture: A Culture-Independent Approach
Source: Front Microbiol. 2022 Jan 3;12:799378. doi: 10.3389/fmicb.2021.799378 (PMC8761758; doi:10.3389/fmicb.2021.799378)
Supplement: Supplementary file 1 [file Data_Sheet_1.docx]

**Supplementary Figures and Tables**

**1.1. Supplementary Figures**

**
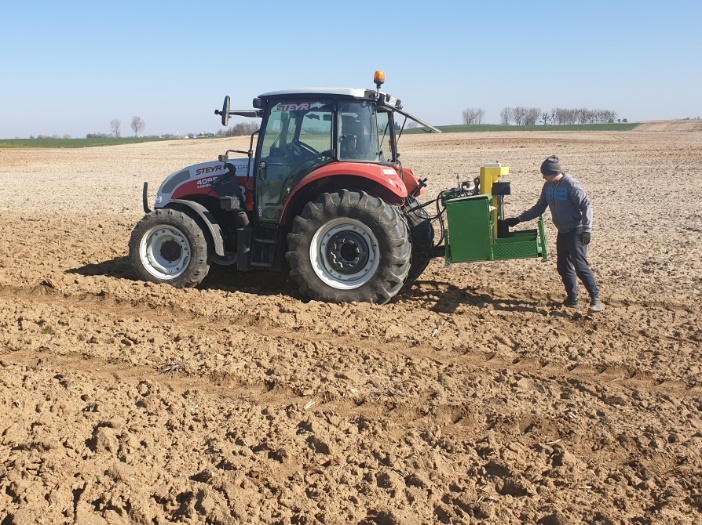

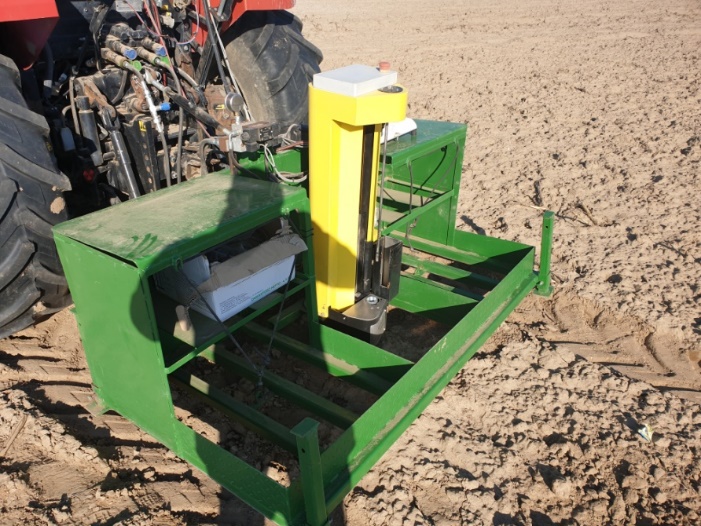

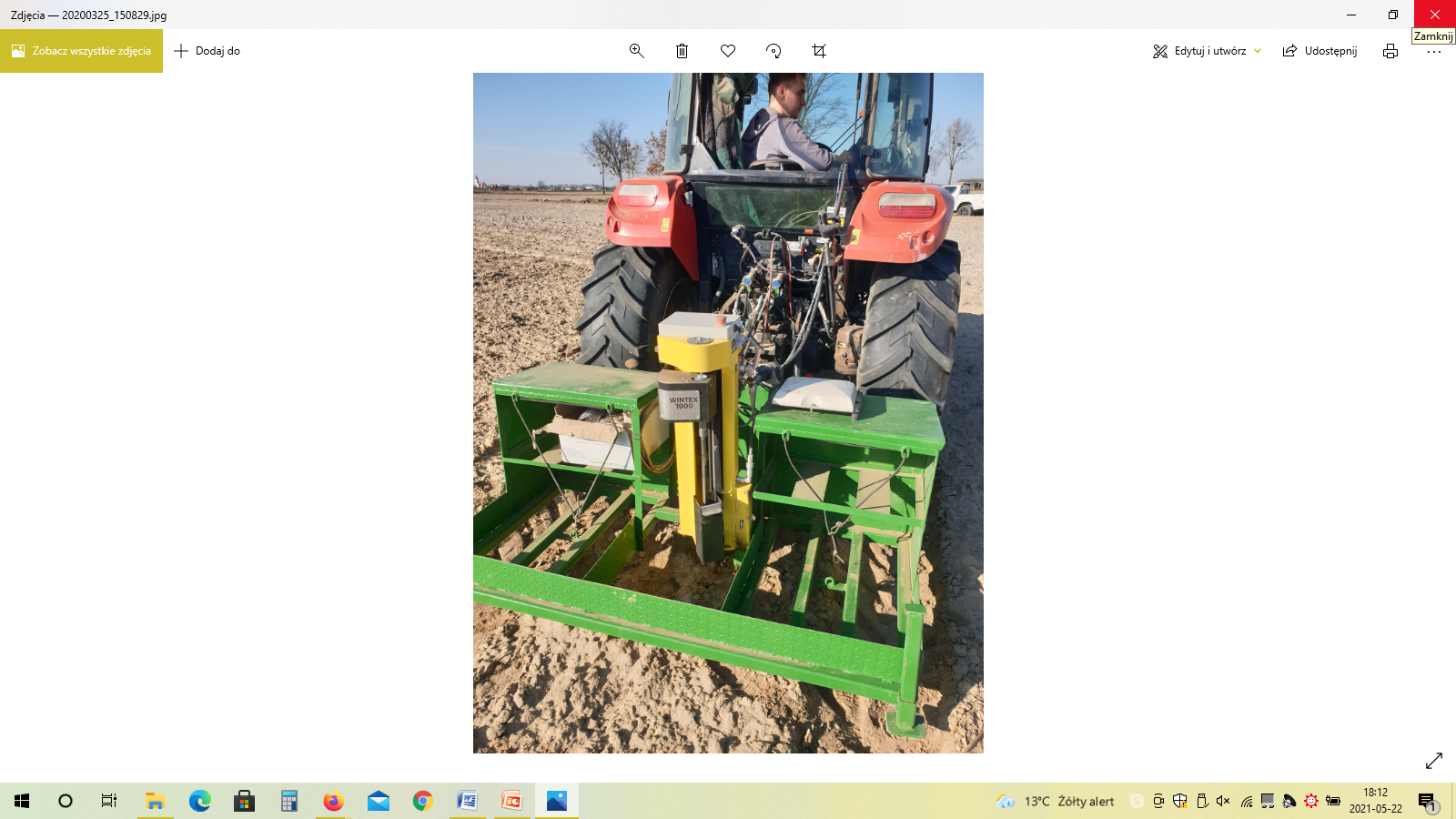

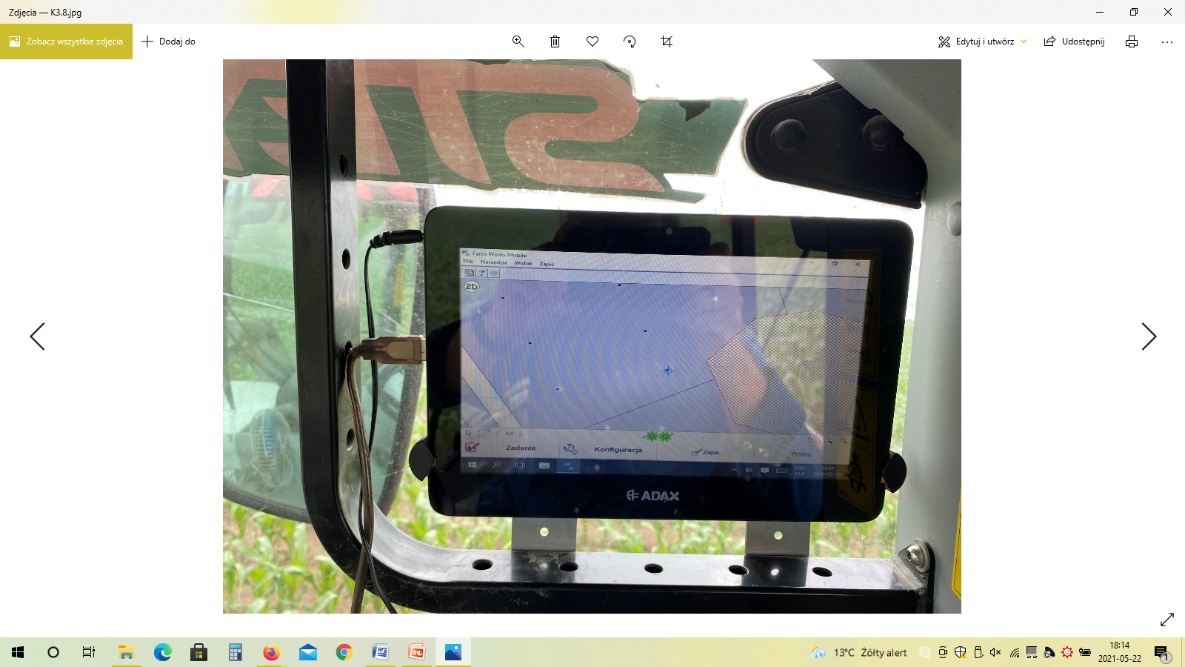
**

**Supplementary Figure S1.** Automatic Egner cane and GPS system for representative sampling for laboratory analysis (photo: A. Slomczewski)

**Supplementary Figure S2.** Rarefaction curves for the studied soil rasters based on the taxa (S) number.

**Supplementary Figure S3.** Subdominant (>2%) fungal genera in the studied rasters of K20 field under intercropping mixture and K21 field under maize monoculture in spring, summer and autumn seasons.


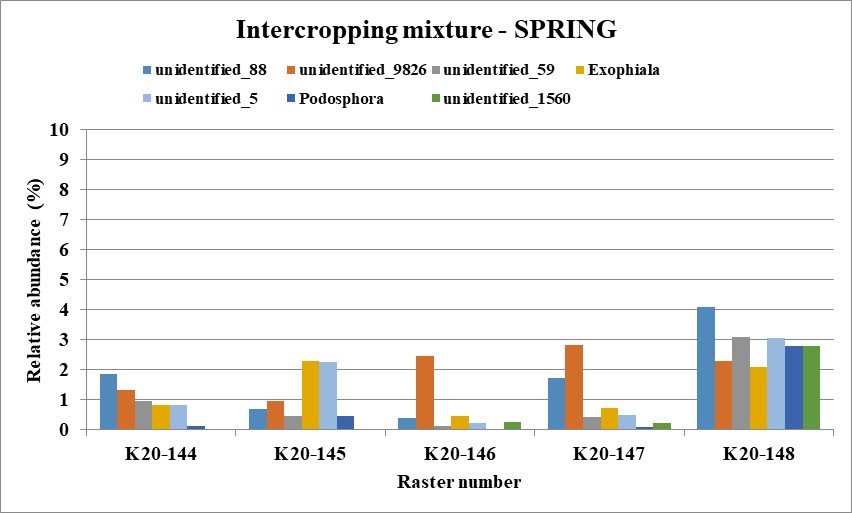

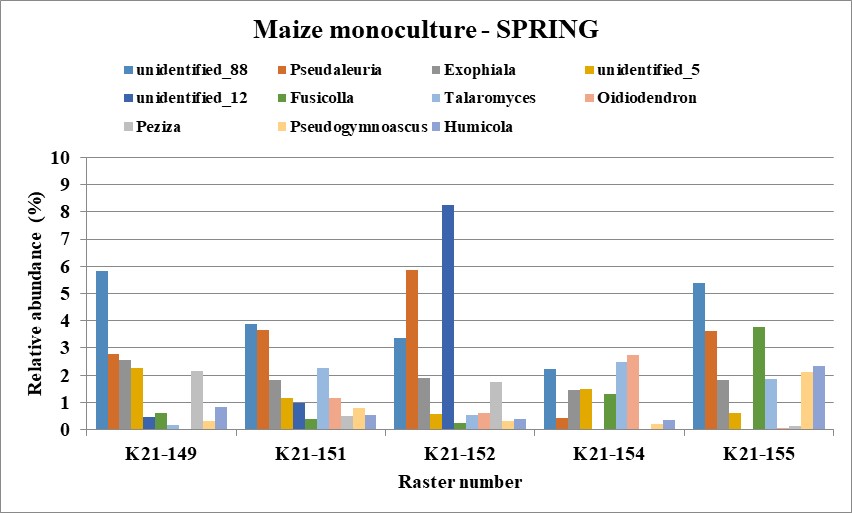

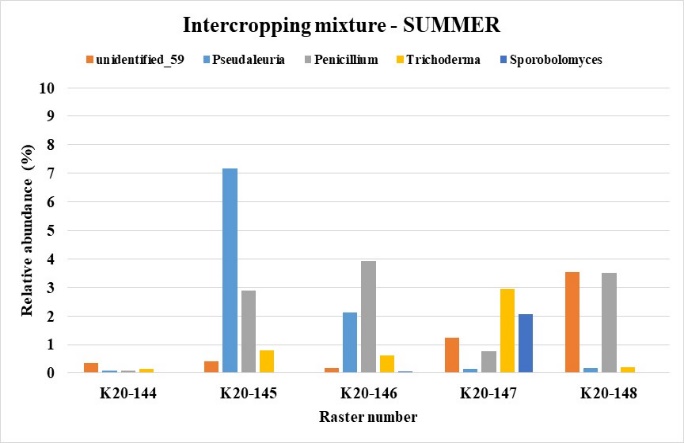

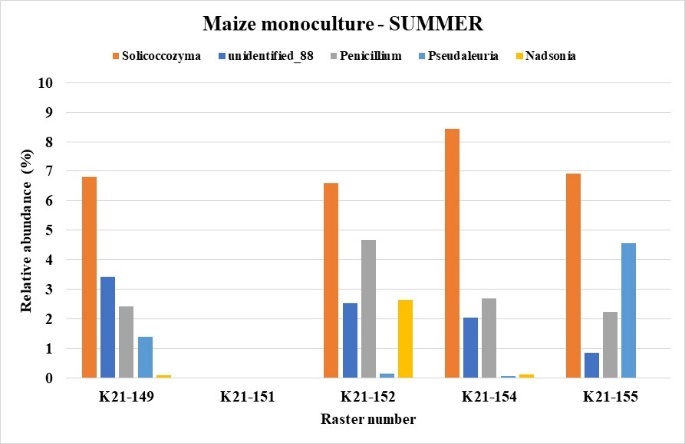

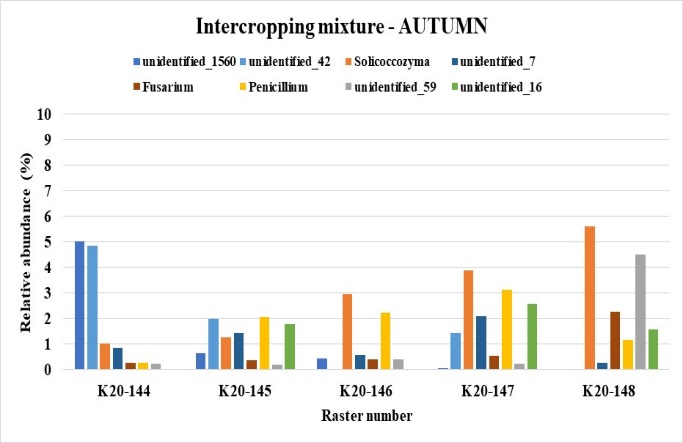

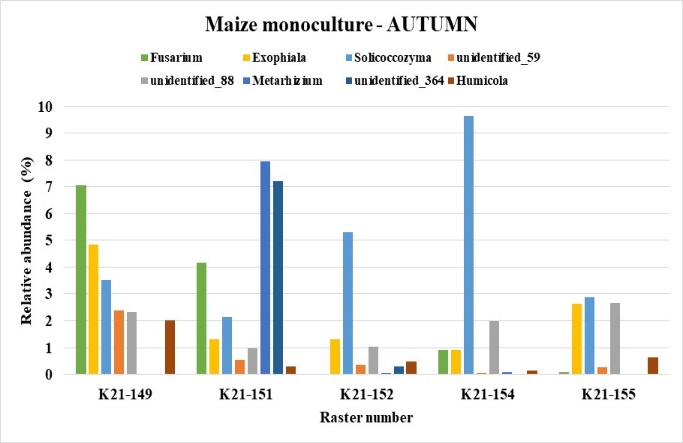


**Supplementary Figure S4.** Accompanying (>1%) fungal genera in the studied rasters of K20 field under intercropping mixture and K21 field under maize monoculture in spring, summer and autumn seasons


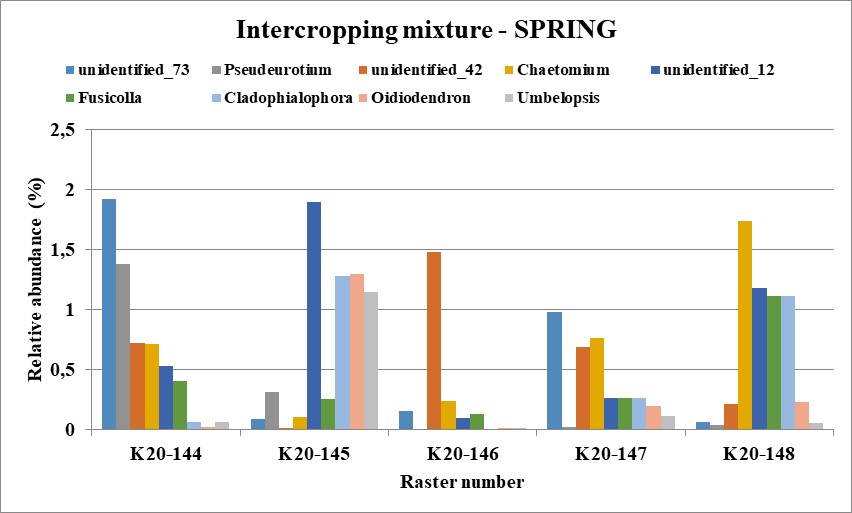

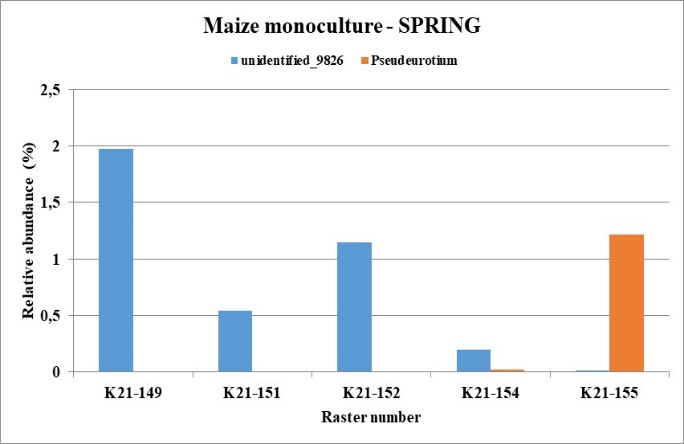

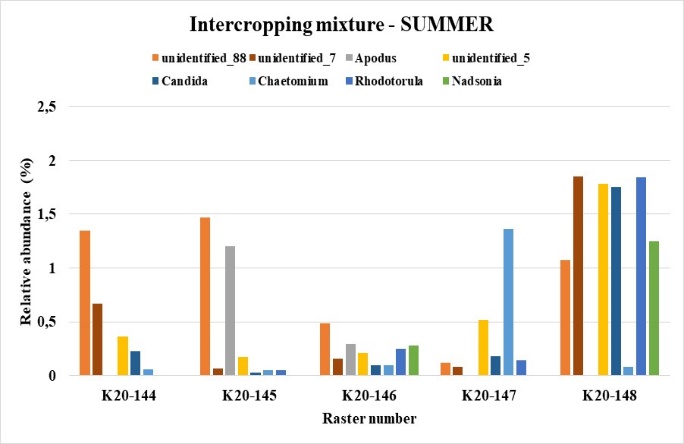

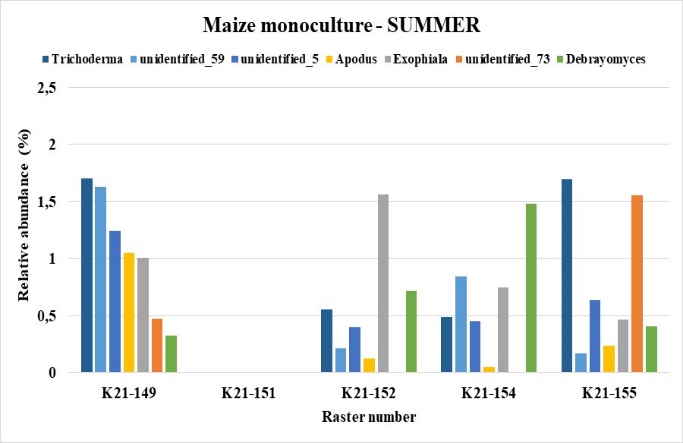

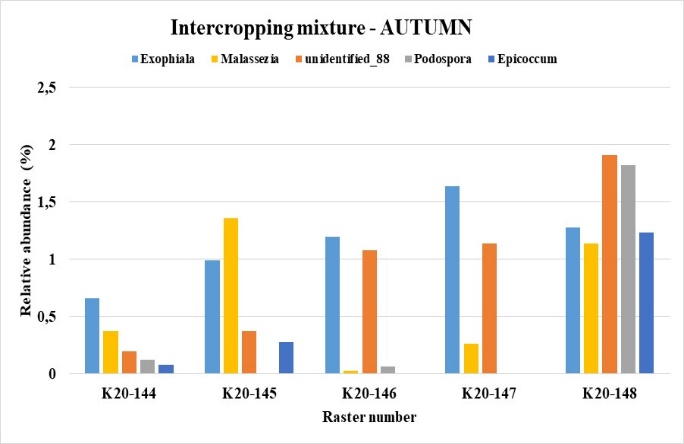

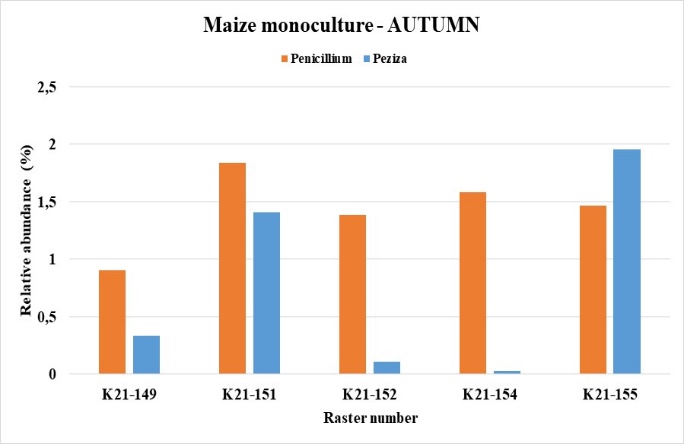


**1.2. Supplementary Tables**

**Table S1.** The basic chemical properties of the studied soils at different seasons: K20 - intercropping mixture and K21 - maize monoculture (mean values of three replicates ± standard deviation level). Small letters in the upper index mean significant differences in the parameters between studied soil rasters (Tukey’s mean separation test, *p*<0.05, n=3).

| **Nr of raster** | **pH_(H2O)_** | **Eh (mV)** | **TOC (%)** | **Moisture (%)** |
| --- | --- | --- | --- | --- |
| **SPRING** | | | | |
| K20-144 | 6.07^a^±0.04 | 509.23^b^±0.12 | 0.71^b^±0.01 | 4.59^a^±0.07 |
| K20-145 | 6.07^a^±0.03 | 526.50^c^±0.85 | 0.82^b^±0.04 | 5.89^b^±0.11 |
| K20-146 | 7.90^c^±0.03 | 493.60^a^±0.08 | 0.61^a^±0.03 | 7.56^c^±0.05 |
| K20-147 | 6.21^a^±0.03 | 514.30^c^±0.16 | 1.16^c^±0.04 | 9.53^d^±0.08 |
| K20-148 | 6.07^a^±0.04 | 525.60^c^±0.21 | 1.34^d^±0.03 | 8.92^e^±0.06 |
| K21-149 | 6.02^a^±0.01 | 514.70^b^±0.04 | 0.52^e^±0.03 | 6.20^b^±0.03 |
| K21-151 | 6.40^b^±0.01 | 509.60^b^±1.60 | 0.59^e^±0.03 | 7.36^c^±0.06 |
| K21-152 | 5.82^d^±0.04 | 520.20^c^±0.08 | 0.56^e^±0.01 | 8.51^e^±0.12 |
| K21-154 | 6.83^b^±0.02 | 507.00^b^±0.16 | 0.67^a^±0.05 | 7.40^c^±0.06 |
| K21-155 | 7.93^c^±0.03 | 499.70**^a^**±0.21 | 1.02^c^±0.02 | 8.41^e^±0.05 |
| **SUMMER** | | | | |
| K20-144 | 6.63^b^±0.05 | 534.40^d^±0.20 | 0.85^b^±0.02 | 10.26^f^±0.03 |
| K20-145 | 6.20^a^±0.05 | 534.33^d^±0.26 | 0.79^b^±0.02 | 7.70^c^±0.06 |
| K20-146 | 5.95^d^±0.01 | 533.86^d^±0.25 | 0.77^b^±0.03 | 9.13^e^±0.07 |
| K20-147 | 6.29^a^±0.03 | 527.07^c^±0.20 | 0.77^b^±0.04 | 10.92^f^±0.04 |
| K20-148 | 5.70^d^±0.02 | 534.57^d^±0.05 | 1.14^c^±0.04 | 14.64^g^±0.08 |
| K21-149 | 5.31^e^±0.02 | 561.53^e^±0.70 | 0.71^b^±0.05 | 10.24^f^±0.03 |
| K21-151 | 5.44^e^±0.01 | 567.60^e^±0.17 | 0.45^e^±0.01 | 9.09^e^±0.08 |
| K21-152 | 5.46^e^±0.01 | 568.03^e^±0.59 | 0.62^a^±0.04 | 8.84^e^±0.06 |
| K21-154 | 6.08^a^±0.02 | 542.67^d^±0.37 | 0.77^b^±0.03 | 10.73^f^±0.09 |
| K21-155 | 6.60^b^±0.04 | 537.73^d^±0.95 | 0.70^b^±0.04 | 10.44^f^±0.05 |
| **AUTUMN** | | | | |
| K20-144 | 6.17^a^±0.03 | 491.07^a^±0.28 | 0.58^e^±0.02 | 13.05^g^±0.06 |
| K20-145 | 6.60^b^±0.01 | 417.73^f^±3.02 | 0.52^e^±0.03 | 10.88^f^±0.08 |
| K20-146 | 6.94^c^±0.01 | 424.83^f^±0.12 | 0.42^e^±0.01 | 12.23^h^±0.09 |
| K20-147 | 6.61^b^±0.03 | 469.67^a^±0.25 | 0.51^e^±0.05 | 12.01^h^±0.11 |
| K20-148 | 5.99^a^±0.16 | 499.13^a^±0.76 | 0.87^b^±0.01 | 16.32^i^±0.04 |
| K21-149 | 5.99^a^±0.02 | 496.53^a^±0.33 | 0.23^f^±0.02 | 11.87^f^±0.06 |
| K21-151 | 5.84^d^±0.01 | 515.30^c^±0.21 | 0.35^f^±0.01 | 9.66^e^±0.03 |
| K21-152 | 6.33^b^±0.01 | 506.57^b^±0.23 | 0.36^f^±0.02 | 9.61e±0.05 |
| K21-154 | 6.55^b^±0.02 | 484.07^a^±0.17 | 0.39^e^±0.03 | 13.35^g^±0.02 |
| K21-155 | 6.99^c^±0.04 | 426.17^f^±0.12 | 0.40^e^±0.03 | 11.88^f^±0.03 |

**Table S2.** DNA concentration and purity in the studied soils at different seasons: K20 - intercropping mixture and K21 - maize monoculture (mean values of three replicates ± standard deviation level)

| **Nr of raster** | **DNA content [µg·ml^-1^]** | **A260/280**  **ratio** | **A260/230 ratio** |
| --- | --- | --- | --- |
| **SPRING** | | |  |
| K20-144 | 9.05±2.33 | 1.54±0.11 | 0.50±0.01 |
| K20-145 | 13.90±2.05 | 1.71±0.16 | 0.54±0.13 |
| K20-146 | 8.75±1.91 | 1.66±0.03 | 0.61±0.08 |
| K20-147 | 11.75±2.59 | 1.62±0.05 | 0.60±0.04 |
| K20-148 | 17.70±3.82 | 1.57±0.04 | 0.53±0.02 |
| K21-149 | 13.30±1.57 | 1.52±0.16 | 0.48±0.06 |
| K21-151 | 8.10±1.54 | 1.62±0.09 | 0.49±0.02 |
| K21-152 | 13.85±1.10 | 1.51±0.18 | 0.48±0.01 |
| K21-154 | 13.84±2.60 | 1.62±0.04 | 0.56±0.00 |
| K21-155 | 18.10±1.08 | 1.72±0.04 | 0.86±0.02 |
| **SUMMER** | | |  |
| K20-144 | 16.04±0.24 | 1.60±0.04 | 0.46±0.01 |
| K20-145 | 14.78±0.25 | 1.63±0.04 | 0.42±0.01 |
| K20-146 | 8.61±0.15 | 1.59±0.05 | 0.32±0.01 |
| K20-147 | 20.89±0.86 | 1.57±0.09 | 0.47±0.01 |
| K20-148 | 19.66±0.17 | 1.63±0.02 | 0.55±0.01 |
| K21-149 | 18.99±0.58 | 1.62±0.10 | 0.47±0.01 |
| K21-151 | 9.63±0.33 | 1.67±0.06 | 0.35±0.01 |
| K21-152 | 9.84±0.12 | 1.52±0.04 | 0.39±0.01 |
| K21-154 | 12.25±0.19 | 1.53±0.04 | 0.44±0.01 |
| K21-155 | 12.60±0.29 | 1.59±0.04 | 0.44±0.01 |
| **AUTUMN** | | |  |
| K20-144 | 4.042±2.43 | 1.76±0.07 | 1.55±0.22 |
| K20-145 | 3.57±0.67 | 1.72±0.12 | 1.00±0.09 |
| K20-146 | 2.38±0.95 | 1.60±0.09 | 0.75±0.16 |
| K20-147 | 1.66±1.11 | 1.48±0.05 | 0.65±0.03 |
| K20-148 | 3.08±0.04 | 1.81±0.02 | 0.74±0.15 |
| K21-149 | 2.44±0.25 | 2.06±0.06 | 0.48±0.25 |
| K21-151 | 10.19±0.54 | 1.44±0.05 | 0.55±0.14 |
| K21-152 | 10.72±0.75 | 1.88±0.03 | 0.49±0.06 |
| K21-154 | 5.67±0.69 | 1.67±0.19 | 0.73±0.03 |
| K21-155 | 8.45±0.84 | 1.72±0.10 | 0.80±033 |
